# Supplementary material for: Immunotherapy targeting different immune compartments in combination with radiation therapy induces regression of resistant tumors
Source: Nat Commun. 2023 Aug 24;14:5146. doi: 10.1038/s41467-023-40844-3 (PMC10449830; doi:10.1038/s41467-023-40844-3)
Supplement: Supplementary file 1 — Supplementary Information [file 41467_2023_40844_MOESM1_ESM.pdf]

SUPPLEMENTARY FIGURES

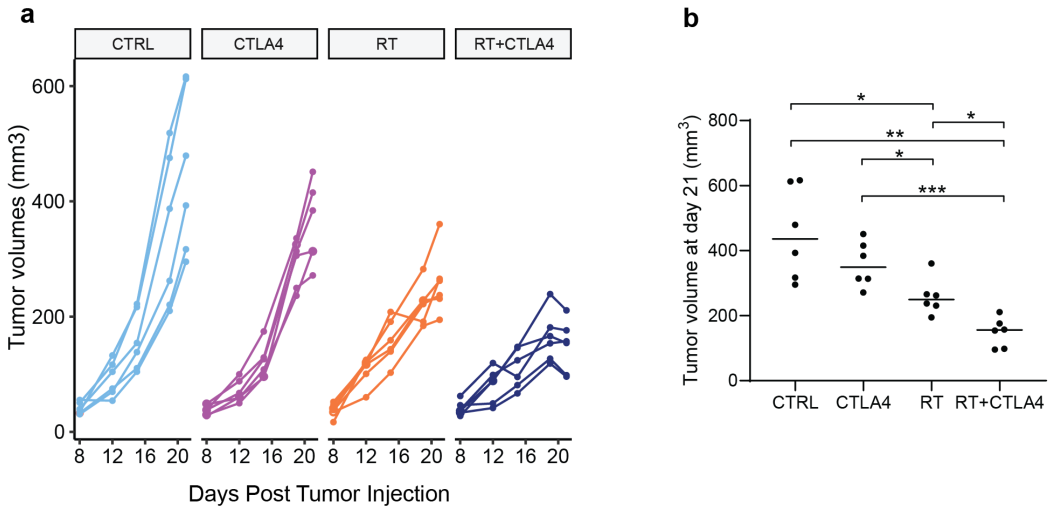

Supplementary Figure 1: Data related to Figure 1 a and b.
BALB/c mice implanted with 4T1 tumors were treated following the schedules previously described, n=6 biologically independent mice/group (a,b), tumors were collected at day 22 for RNA and TCR sequencing analysis. **(a)** Individual tumor growth curves **(b)** Tumor volume at day of sacrifice (day 21). Horizontal lines show medians of each group. *, **, and *** indicate p-values <0.05, 0.01, and 0.001 respectively, calculated using a two-sided t-test. Source data and exact p values are provided in the Source Data file.

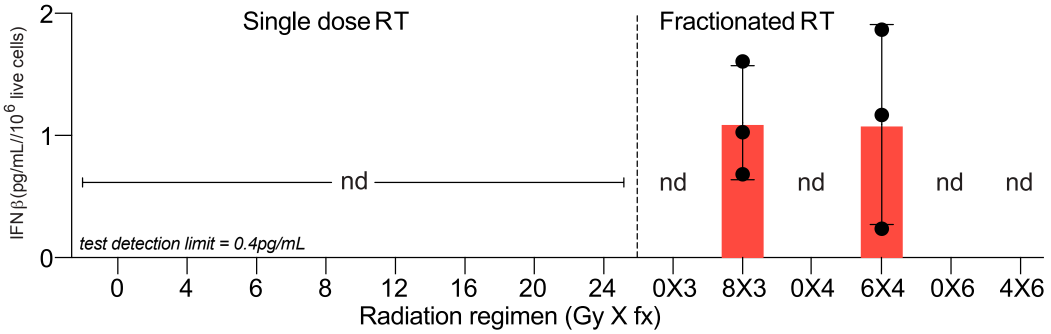

Supplementary Figure 2: Interferon-β (IFNβ) secretion by 4T1 cells following in vitro irradiation. IFN β concentration was measured by ELISA in cell supernatants collected 24 hours after the last radiation dose, each dot represents a biological triplicate. Data are shown as mean ± SD. Source data are provided as a Source Data file.

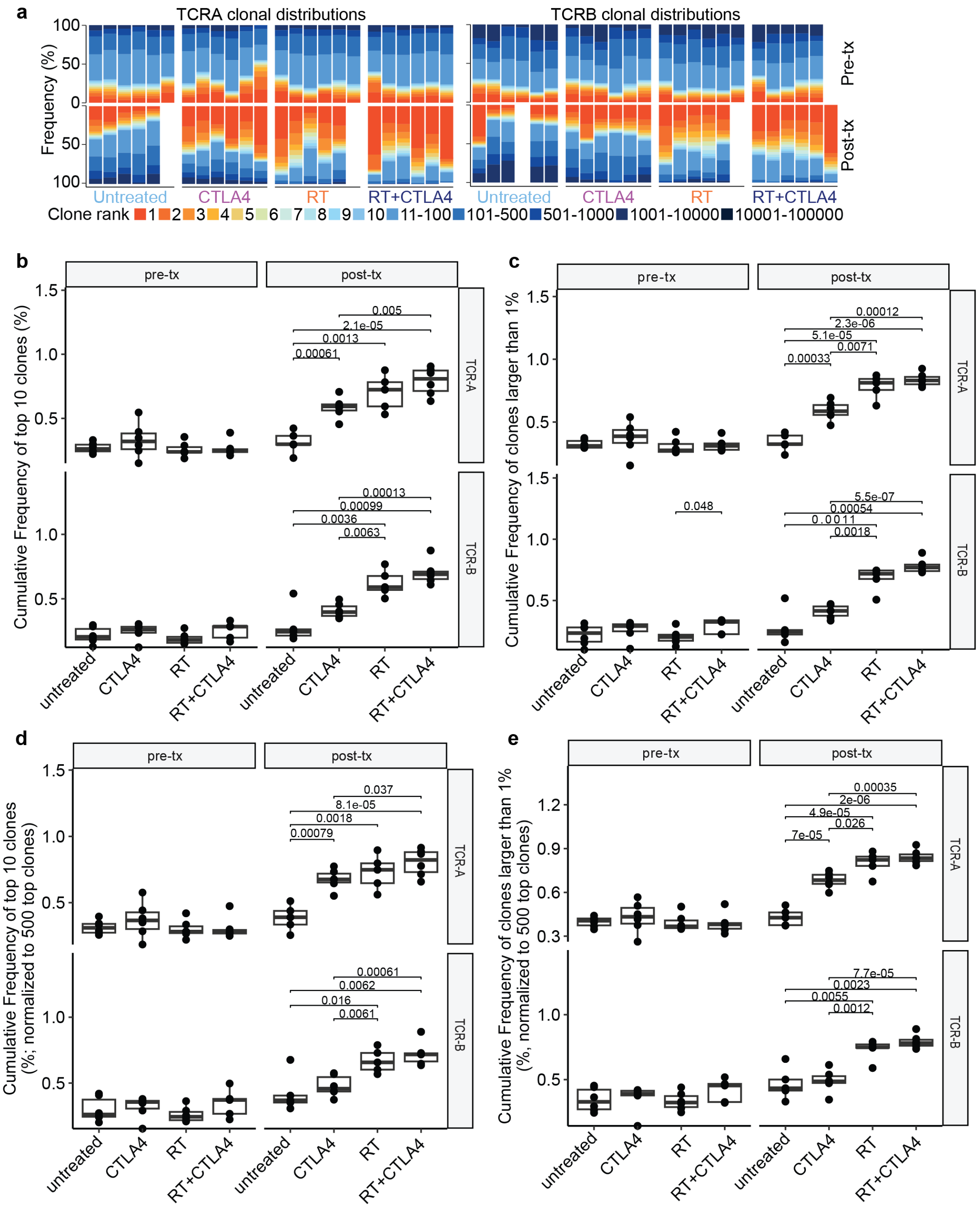

Supplementary Figure 3: Clonal distributions of TCR repertoires. Data related to Figure 1. **(a)** Each column represents a mouse, and upper and bottom bars represent pre- (pre-tx) and post-treatment (post-tx) tumors, respectively. Color indicates clone rank. In some animals one of the samples did not meet the QC standards and is not included. **(b-c)** Cumulative frequency of the top 10 clones **(b)** and of any clone with a frequency above 1% for each treatment group pre- and

post-treatment **(c)**. **(d-e)** Normalized cumulative frequency of the top 10 clones **(d)** and of any clone with a frequency above 1% **(e)**. The top 500 clonotypes were used to normalize frequencies. Statistical significance was calculated using a two-sided t-test, n=6 biologically independent mice per group. Data is visualized using the ggplot2::geom_boxplot function with default settings (center line=median value, hinges=25th and 75th percentile, upper and lower whisker extends from the hinge to the largest and smaller value no further than 1.5 * inter-quartile range (IQR), individual values are shown as filled circles). Source data are provided as a Source Data file.

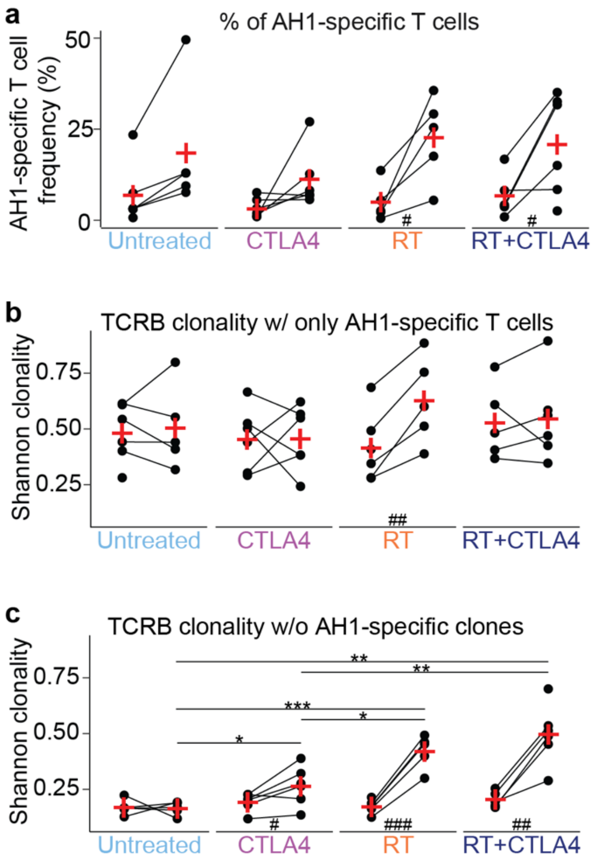

Supplementary Figure 4: Clonality of TCRB repertoires segregated by AH1 reactivity. **Related to Figure 1.**

(a) Frequency of AH1-specific T cells in pre- and post-tx tumors calculated using previously published data on AH1-dextramer$^+$ CD8$^+$ T cells (*Rudqvist NP, et al. Cancer Immunol Res 6, 139-150, 2018*) and single cell VDJ and feature barcoding data from this paper. **(b)** Clonality of AH1-specific TCRB repertoires, and **(c)** clonality of AH1-unrelated TCRB repertoires. n=6 biologically independent mice per group. Red crosses indicate mean. *, **, ***, and #, ##, and ###, indicate p-values < 0.05, 0.01, and 0.001 for pairwise and two-sided paired t-tests, respectively. Source data and exact p values are provided in the Source Data file.

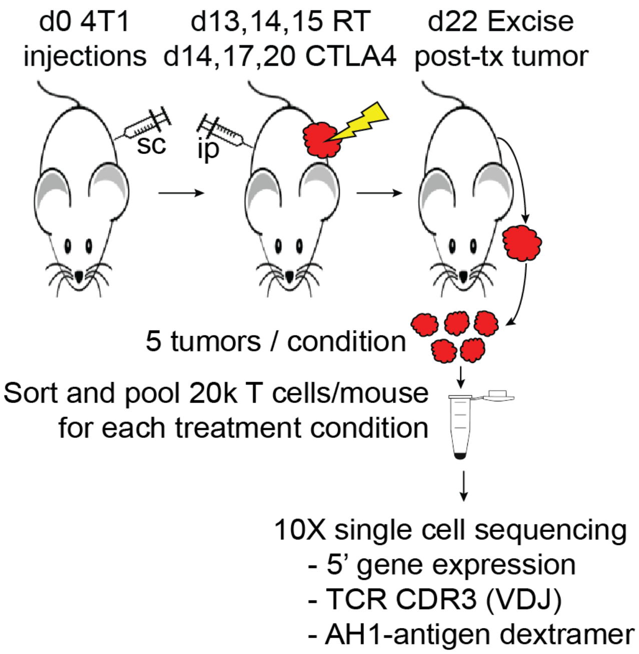

Supplementary Figure 5: Diagram of single cell sequencing experiments, related to Figure 3.

Single positive $CD3^+$ cells from 4T1 tumors were processed using single cell sequencing (RNA/TCR/AH1-dCODE dextramer).

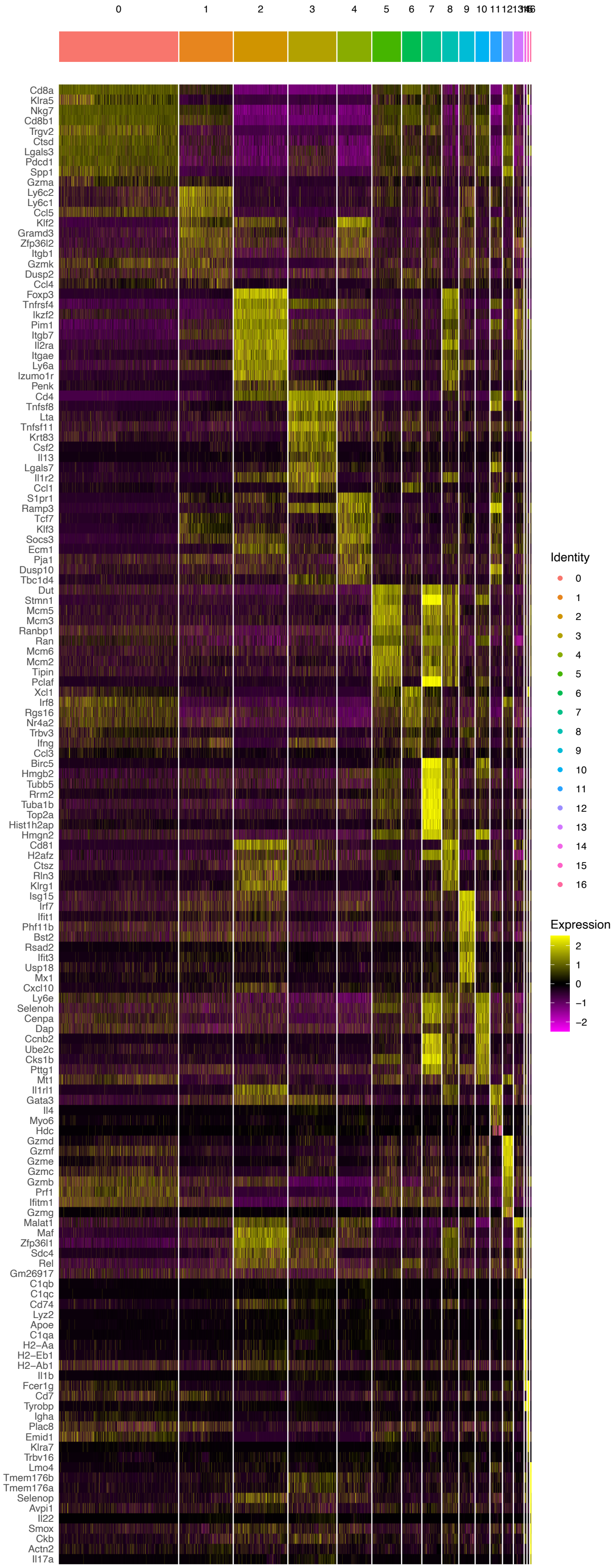
0
1
2
3
4
5
6
7
8
9
10
11
12
Cd8a
Klra5
Nkg7
Cd8b1
Trgv2
Ctsd
Lgals3
Pdcd1
Spp1
Gzma
Ly6c2
Ly6c1
Ccl5
Klf2
Gramd3
Zfp36l2
Itgb1
Gzmk
Dusp2
Ccl4
Foxp3
Tnfrsf4
Ikzf2
Pim1
Itgb7
Il2ra
Itgae
Ly6a
Izumo1r
Penk
Cd4
Tnfsf8
Lta
Tnfsf11
Krt83
Csf2
Il13
Lgals7
Il1r2
Ccl1
S1pr1
Ramp3
Tcf7
Klf3
Socs3
Ecm1
Pja1
Dusp10
Tbc1d4
Dut
Stmn1
Mcm5
Mcm3
Ranbp1
Ran
Mcm6
Mcm2
Tipin
Pclaf
Xcl1
Irf8
Rgs16
Nr4a2
Trbv3
Ifng
Ccl3
Birc5
Hmgb2
Tubb5
Rrm2
Tuba1b
Top2a
Hist1h2ap
Hmgn2
Cd81
H2afz
Ctsz
Rln3
Klrg1
Isg15
Irf7
Ifit1
Phf11b
Bst2
Rsad2
Ifit3
Usp18
Mx1
Cxcl10
Ly6e
Selenoh
Cenpa
Dap
Ccnb2
Ube2c
Cks1b
Pttg1
Mt1
Il1rl1
Gata3
Il4
Myo6
Hdc
Gzmd
Gzmf
Gzme
Gzmc
Gzmb
Prf1
Ifitm1
Gzmg
Malat1
Maf
Zfp36l1
Sdc4
Rel
Gm26917
C1qb
C1qc
Cd74
Lyz2
Apoe
C1qa
H2−Aa
H2−Eb1
H2−Ab1
Il1b
Fcer1g
Cd7
Tyrobp
Igha
Plac8
Emid1
Klra7
Trbv16
Lmo4
Tmem176b
Tmem176a
Selenop
Avpi1
Il22
Smox
Ckb
Actn2
Il17a
Identity
0
1
2
3
4
5
6
7
8
9
10
11
12
13
14
15
16
Expression
2
1
0
−1
−2

Supplementary Figure 6: Single cell RNA seq of tumor infiltrating $CD3^+$ T cells: cluster definition; related to Figure 3a.

Heatmap showing differentially expressed genes in each cluster from the experiment in Figure 3. Gene names are indicated on the y-axis and the level of differential expression is indicated by the color.

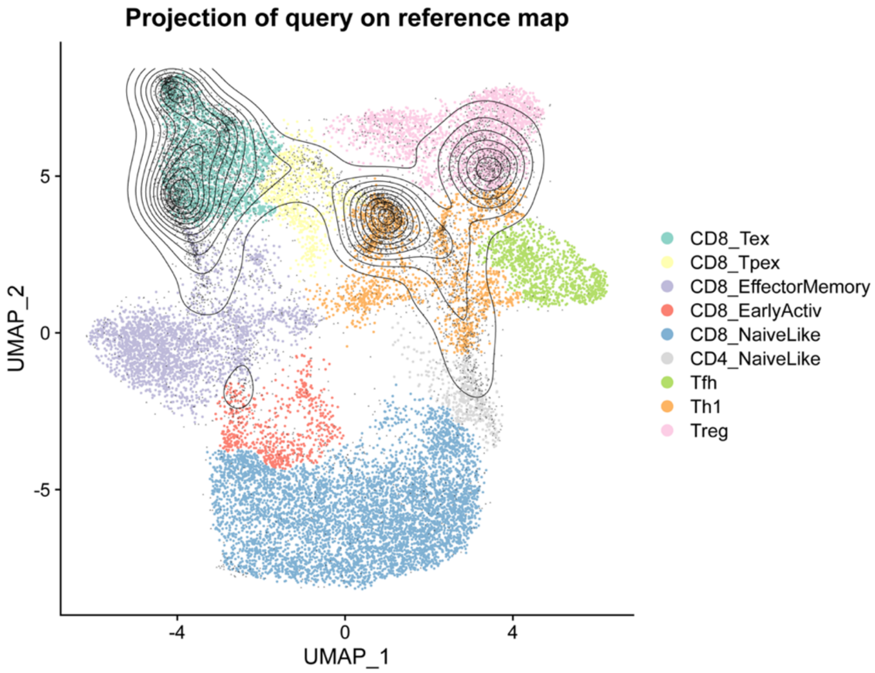

Supplementary Figure 7: ProjecTILs map projection. Related to Figure 3c.
Functional states defined using ProjecTILs project were used to annotate the single cells from digested 4T1 tumors. Colors indicate ProjecTILs functional state. Black dots indicate single cells from 4T1 tumors in this study projected into the UMAP provided by ProjecTILs. Lines indicate density of single cells mapped to the UMAP.

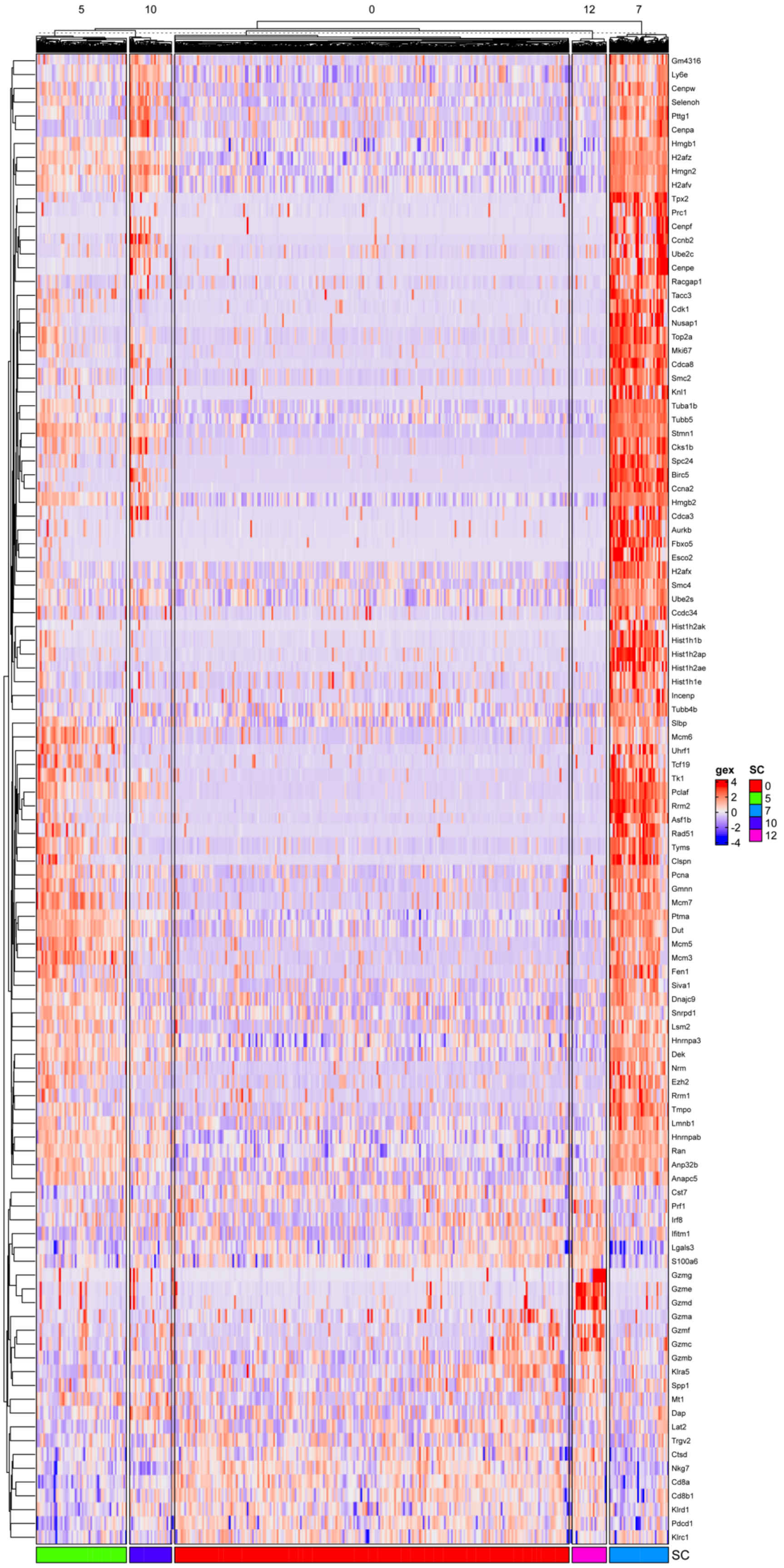
5
10
0
12
7
Gm4316
Ly6e
Cenpw
Selenoh
Pttg1
Cenpa
Hmgb1
H2afz
Hmgn2
H2afv
Tpx2
Prc1
Cenpf
Ccnb2
Ube2c
Cenpe
Racgap1
Tacc3
Cdk1
Nusap1
Top2a
Mki67
Cdca8
Smc2
Knl1
Tuba1b
Tubb5
Stmn1
Cks1b
Spc24
Birc5
Ccna2
Hmgb2
Cdca3
Aurkb
Fbxo5
Esco2
H2afx
Smc4
Ube2s
Ccdc34
Hist1h2ak
Hist1h1b
Hist1h2ap
Hist1h2ae
Hist1h1e
Incenp
Tubb4b
Slbp
Mcm6
Uhrf1
Tcf19
Tk1
Pclaf
Rrm2
Asf1b
Rad51
Tyms
Clspn
Pcna
Gmnn
Mcm7
Ptma
Dut
Mcm5
Mcm3
Fen1
Siva1
Dnajc9
Snrpd1
Lsm2
Hnrnpa3
Dek
Nrm
Ezh2
Rrm1
Tmpo
Lmnb1
Hnrnpab
Ran
Anp32b
Anapc5
Cst7
Prf1
Irf8
Ifitm1
Lgals3
S100a6
Gzmg
Gzme
Gzmd
Gzma
Gzmf
Gzmc
Gzmb
Klra5
Spp1
Mt1
Dap
Lat2
Trgv2
Ctsd
Nkg7
Cd8a
Cd8b1
Klrd1
Pdcd1
Klrc1
SC
gex
4
2
0
-2
-4
SC
0
5
7
10
12

Supplementary Figure 8: Heatmap of differentially expressed genes for CD8 Tex population. Related to figure 3c-i.
Functional state defined using ProjecTILs project. SC indicate single cell cluster defined using Seurat. Differentially expressed genes were determined for each combined functional state plus Seurat cluster, e.g., CD8_Tex in cluster 0 vs. all other cells.

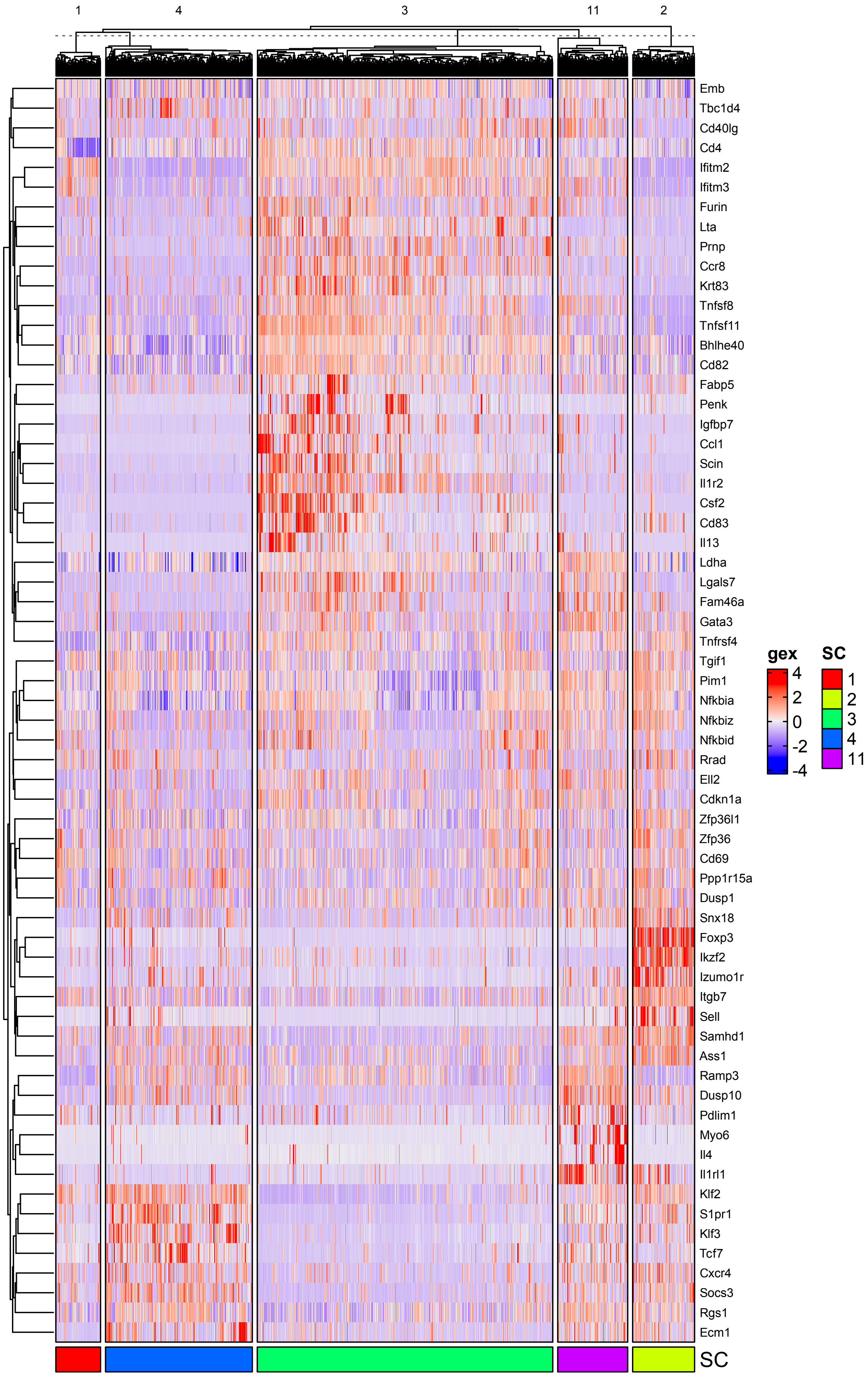

1
4
3
11
2
Emb
Tbc1d4
Cd40lg
Cd4
Ifitm2
Ifitm3
Furin
Lta
Prnp
Ccr8
Krt83
Tnfsf8
Tnfsf11
Bhlhe40
Cd82
Fabp5
Penk
Igfbp7
Ccl1
Scin
Il1r2
Csf2
Cd83
Il13
Ldha
Lgals7
Fam46a
Gata3
Tnfrsf4
Tgif1
Pim1
Nfkbia
Nfkbiz
Nfkbid
Rrad
Ell2
Cdkn1a
Zfp36l1
Zfp36
Cd69
Ppp1r15a
Dusp1
Snx18
Foxp3
Ikzf2
Izumo1r
Itgb7
Sell
Samhd1
Ass1
Ramp3
Dusp10
Pdlim1
Myo6
Il4
Il1rl1
Klf2
S1pr1
Klf3
Tcf7
Cxcr4
Socs3
Rgs1
Ecm1
SC
gex
4
2
0
-2
-4
SC
1
2
3
4
11

Supplementary Figure 9: Heatmap of differentially expressed genes for Th1 population. Related to figure 3c-i.

Functional state defined using ProjecTILs project. SC indicate single cell cluster defined using Seurat. Differentially expressed genes were determined for each combined functional state plus Seurat cluster, e.g., Th1 in cluster 1 vs. all other cells.

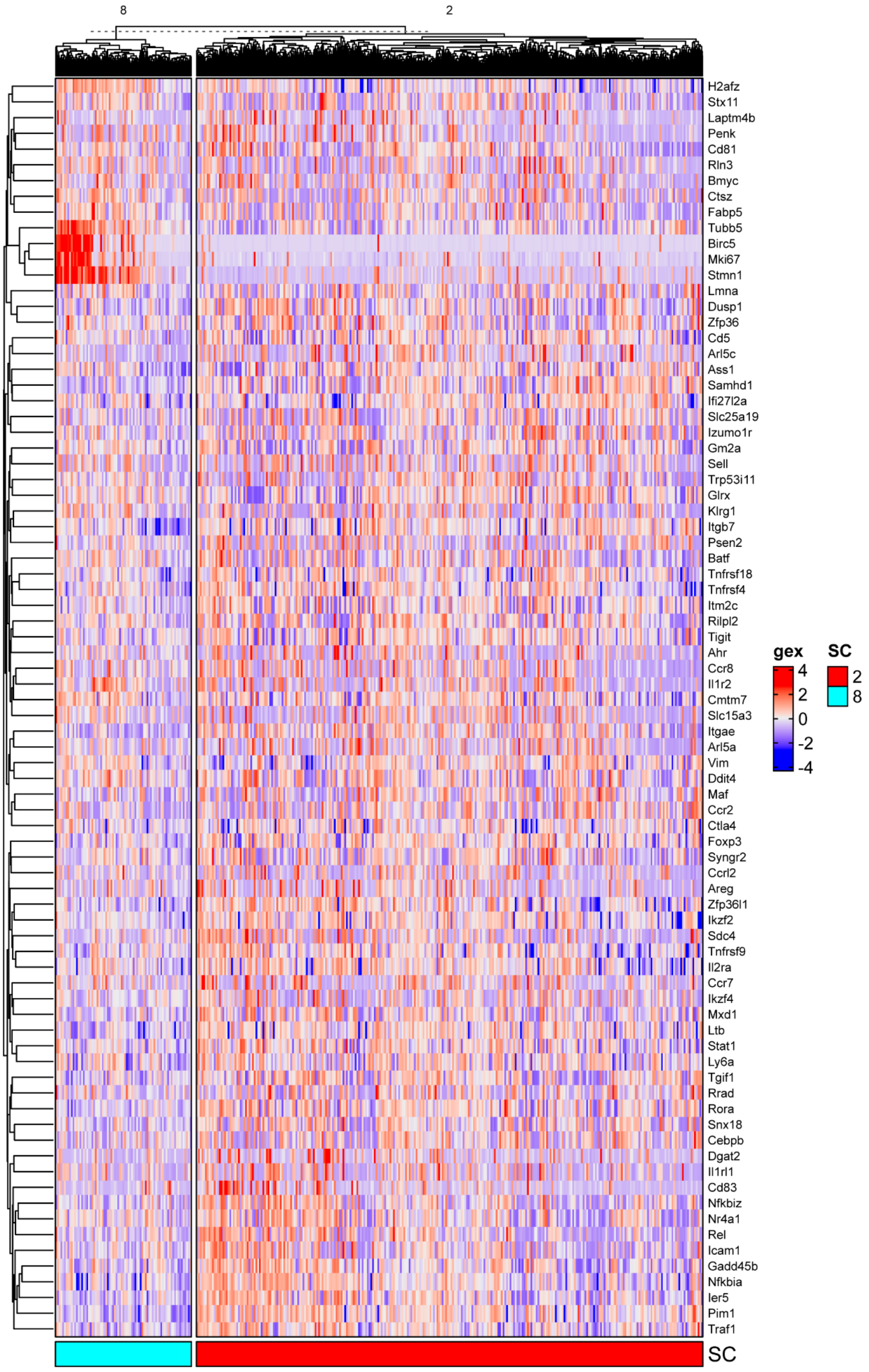

8
2
H2afz
Stx11
Laptm4b
Penk
Cd81
Rln3
Bmyc
Ctsz
Fabp5
Tubb5
Birc5
Mki67
Stmn1
Lmna
Dusp1
Zfp36
Cd5
Arl5c
Ass1
Samhd1
Ifi27l2a
Slc25a19
Izumo1r
Gm2a
Sell
Trp53i11
Glrx
Klrg1
Itgb7
Psen2
Batf
Tnfrsf18
Tnfrsf4
Itm2c
Rilpl2
Tigit
Ahr
Ccr8
Il1r2
Cmtm7
Slc15a3
Itgae
Arl5a
Vim
Ddit4
Maf
Ccr2
Ctla4
Foxp3
Syngr2
Ccrl2
Areg
Zfp36l1
Ikzf2
Sdc4
Tnfrsf9
Il2ra
Ccr7
Ikzf4
Mxd1
Ltb
Stat1
Ly6a
Tgif1
Rrad
Rora
Snx18
Cebpb
Dgat2
Il1rl1
Cd83
Nfkbiz
Nr4a1
Rel
Icam1
Gadd45b
Nfkbia
Ier5
Pim1
Traf1
SC
gex
4
2
0
-2
-4
SC
2
8

Supplementary Figure 10: Heatmap of differentially expressed genes for Treg population. Related to figure 3c-i.

Functional state defined using ProjecTILs project. SC indicate single cell cluster defined using Seurat. Differentially expressed genes were determined for each combined functional state plus Seurat cluster, e.g., Treg in cluster 2 vs. all other cells.

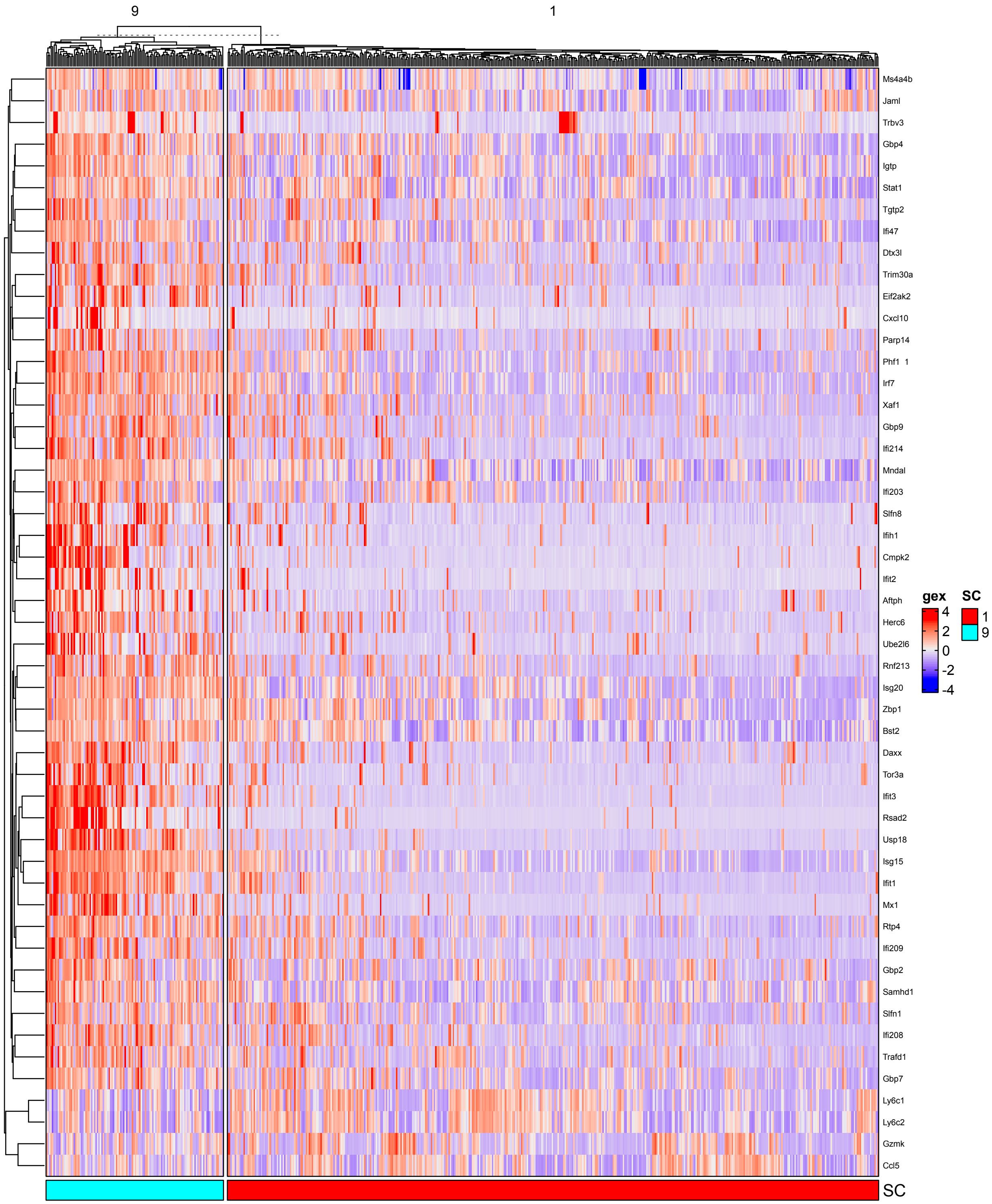

Supplementary Figure 11: Heatmap of differentially expressed genes for CD8 EM population. Related to figure 3c-i.
Functional state defined using ProjecTILs project. SC indicate single cell cluster defined using Seurat. Differentially expressed genes were determined for each combined functional state plus Seurat cluster, e.g., CD8 EM in cluster 1 vs. all other cells.

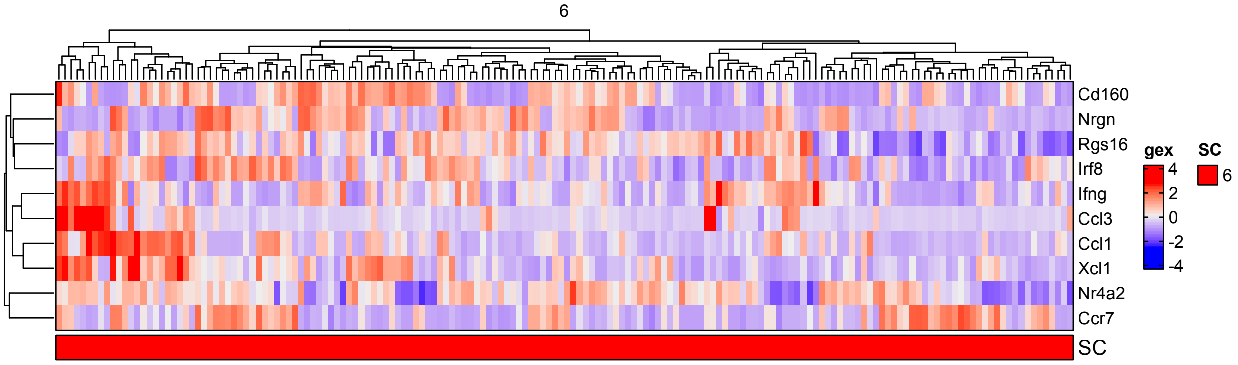

Supplementary Figure 12: Heatmap of differentially expressed genes for CD8 Tpex population. Related to figure 3c-i.
Functional state defined using ProjecTILs project. SC indicate single cell cluster defined using Seurat. Differentially expressed genes were determined for each combined functional state plus Seurat cluster, e.g., CD8 Tpex in cluster 6 vs. all other cells.

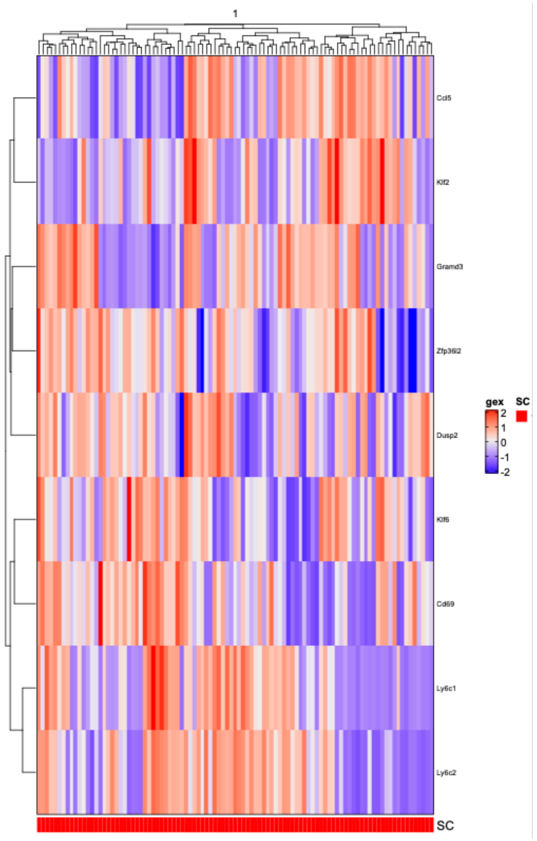

Supplementary Figure 13: Heatmap of differentially expressed genes for CD8 EA population. Related to figure 3c-i.
Functional state defined using ProjecTILs project. SC indicate single cell cluster defined using Seurat. Differentially expressed genes were determined for each combined functional state plus Seurat cluster, e.g., CD8 EA in cluster 1 vs. all other cells.

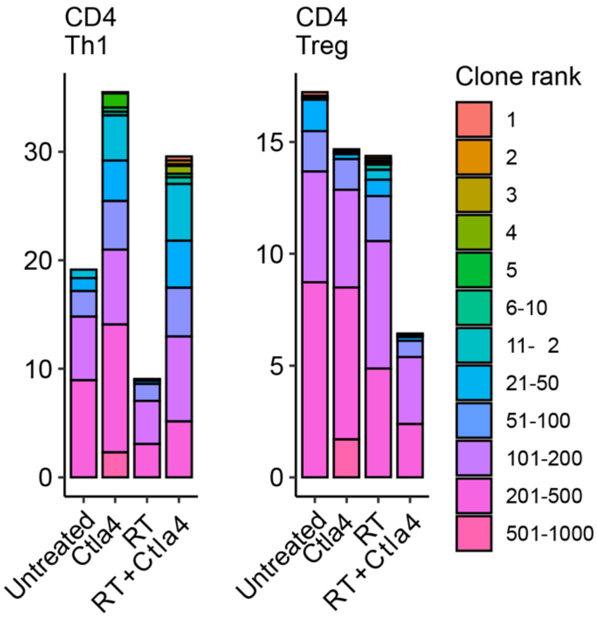

Supplementary Figure 14: Clonal distribution of T cells in CD4 Th1 and Treg subsets. Related to Figure 3h.

T cells were ranked based on frequency and grouped into bins based on rank for each treatment group. Color indicates the rank bin. The total size of bar for each treatment group indicates the proportion (%) of cells associated with each subset. Tumors from 5 mice per treatment group were pooled for scRNAseq analysis.

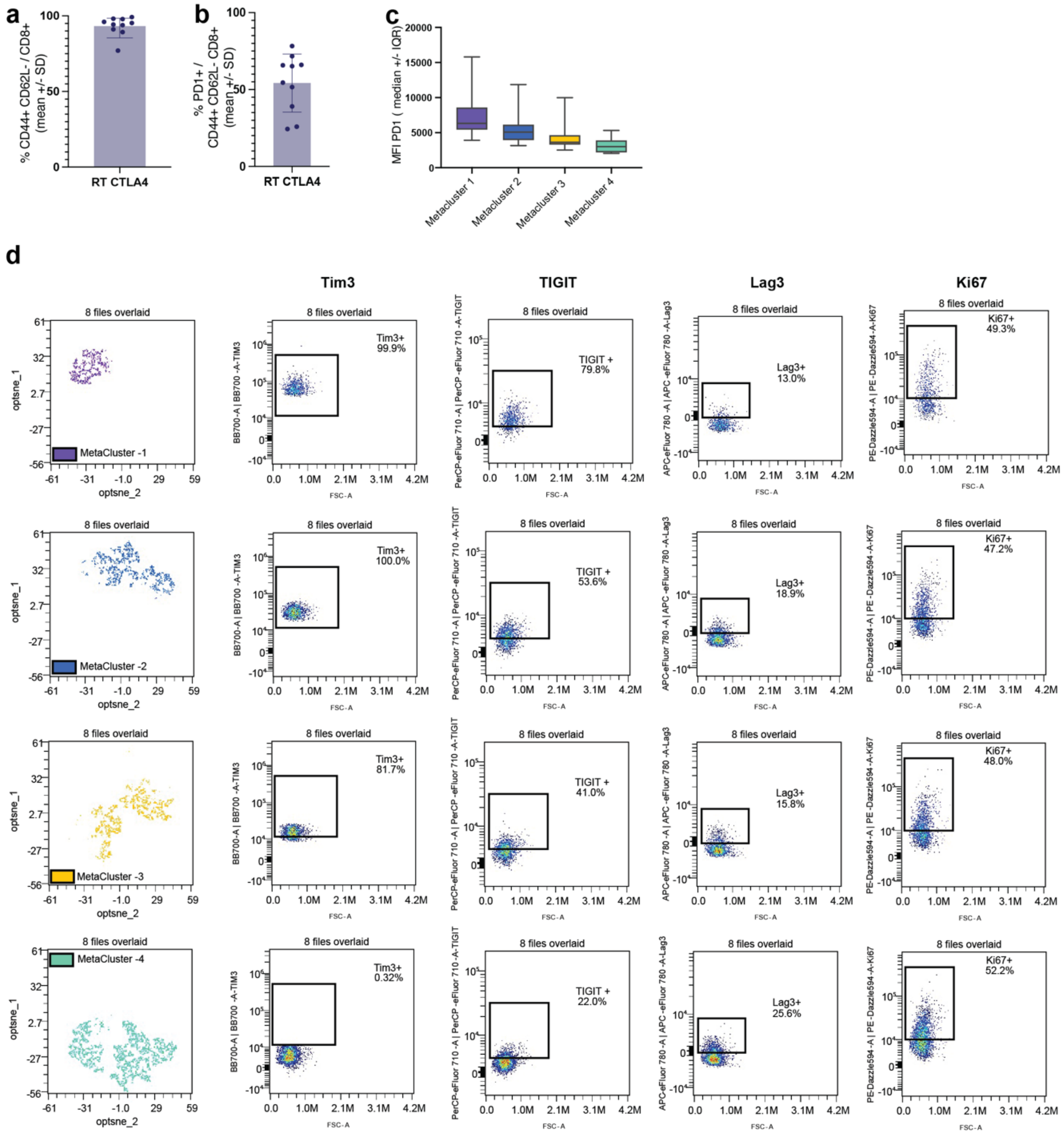

Supplementary Figure 15: CD8^{+} T cell infiltration of 4T1 tumors. Related to figure 5d, e. 4T1 tumors were collected at day 22 after implantation, dissociated and analyzed by flow cytometry. n=10 biologically independent mice per group **(a)** Percentage of antigen experienced T cells (CD44^{+} CD62L^{-}) among CD8 $^{+}$ T cells. **(b)** Percentage of PD1^{+} among antigen experienced CD8^{+} T cells. **(c)** PD1 fluorescence intensity of the 4 metaclusters resolved using Opt-sne and FlowSOM analysis pipelines on PD1^{+} antigen experienced CD8^{+} T cells from RT+CTLA4i treated tumors. Data are visualized as boxplot (center line=median, hinges=25th and 75th percentile, upper and lower whisker indicate min and max values). **(d)** Flow cytometry stainings for Tim3, TIGIT, Lag3 and Ki67 on individual metaclusters. Data are shown as mean ± SD (a, b). Source data are provided as a Source Data file.

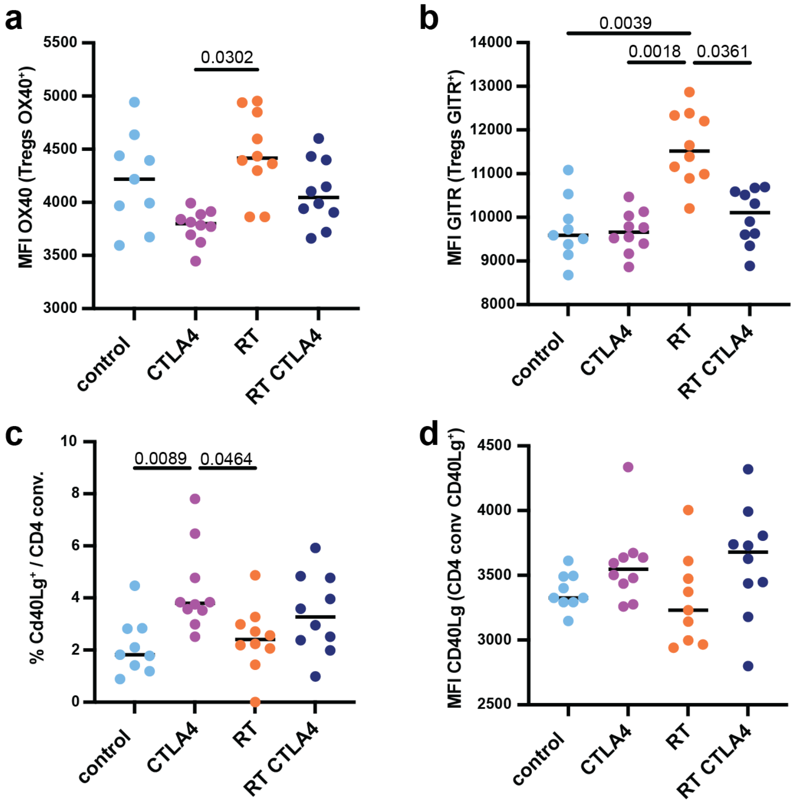

Supplementary Figure 16: Related to Figure 5.
(a) Mean fluorescence intensity (MFI) of OX40^{+} and **(b)** GITR^{+} regulatory CD4^{+} T cells. **(c)** Percentage and **(d)** MFI of CD40Lg positive CD4^{+} T cells. Lines illustrate median and each dot represent an animal, n=10 biologically independent mice per group. Statistical significance was assessed by Kruskal-Wallis test and post-hoc Dunn's test on each panel. Source data are provided in the Source Data file.

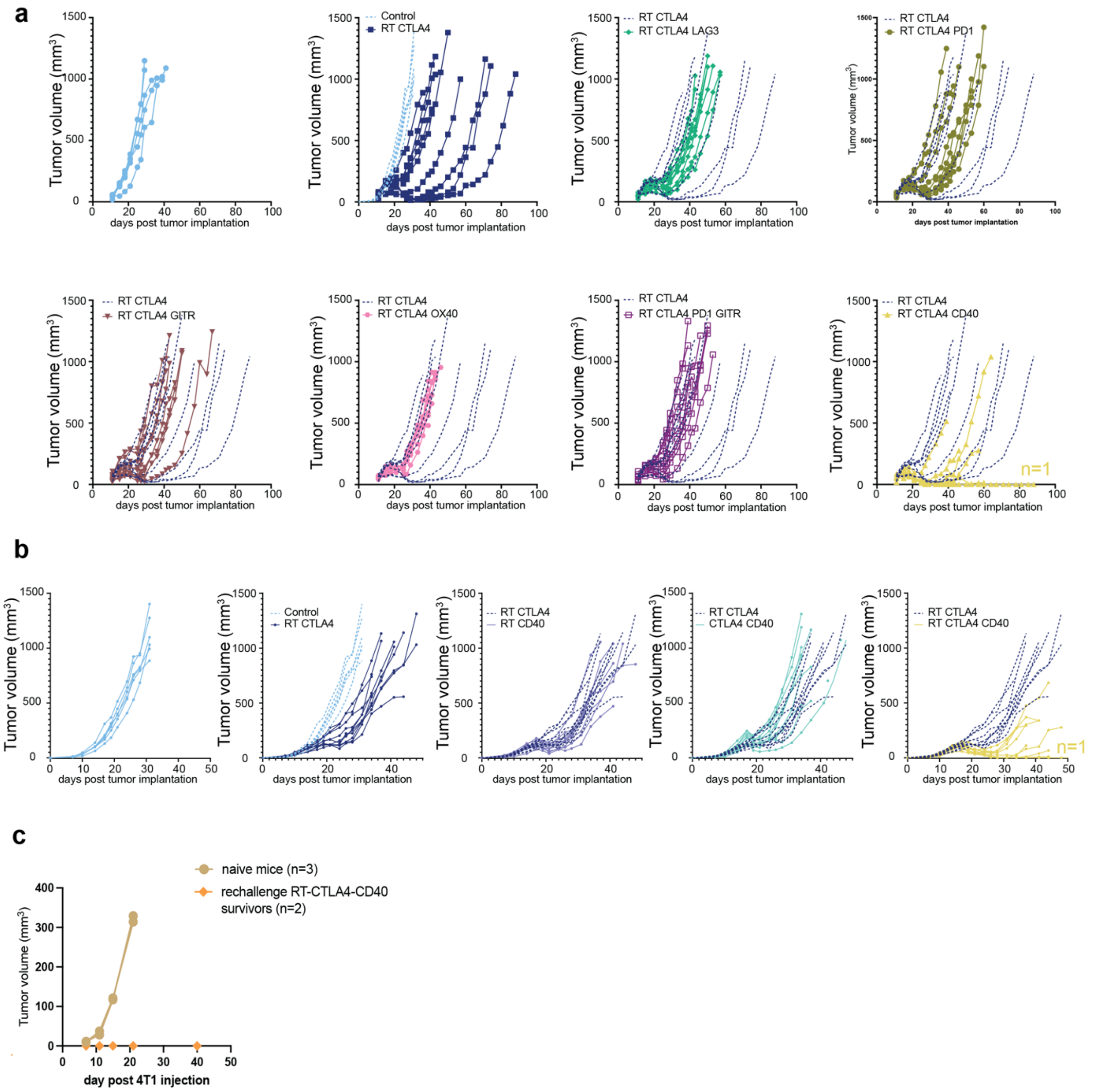

Supplementary Figure 17: Individual tumor growth curves for the experiments presented in Figure 6.

(a) Individual tumor growth curves for mice treated with combination therapies as presented in figure 6a. Control (light blue) or RT CTLA4i (dark blue) are provided in dotted lines on all panels as a comparison. (n=9 biologically independent mice per treatment group, n=5 for control group) **(b)** Individual tumor growth curves for mice treated with combination therapies as presented in figure 6d. Control (light blue) or RT CTLA4i (dark blue) are provided in dotted lines in all graphs as a comparison. Number mice experiencing complete tumor regression are indicated on the graphs. (n=10 per group – 5 for control group) **(c)** Mice with maintained complete response (MCR) from the experiments presented in Figure 6 (c and f) were rechallenged with 4T1 cells on the opposite flank (n=2) together with naïve mice (n=3). Individual tumor growth curves are shown. Source data are provided as a Source Data file.

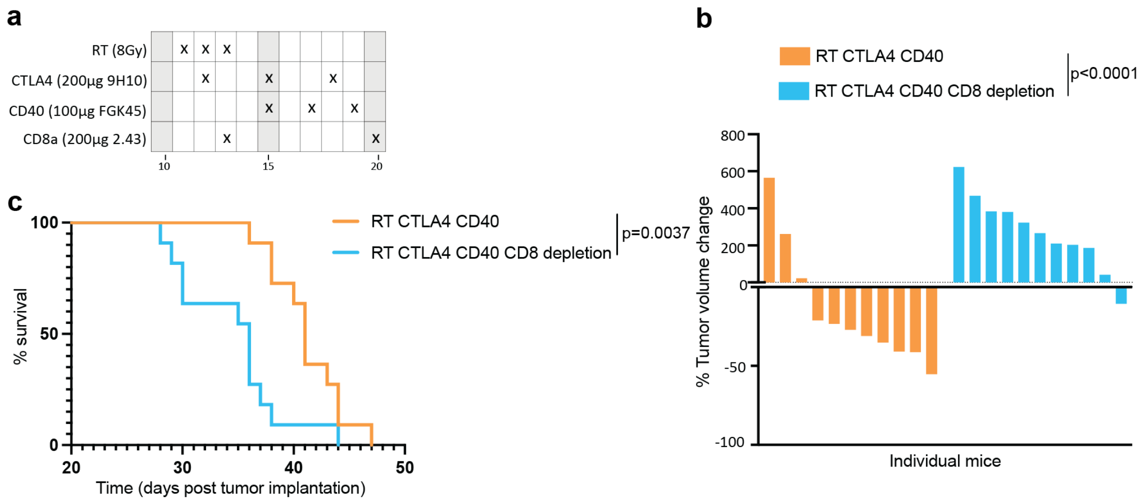

Supplementary Figure 18: Depletion of CD8 T cells abrogates the effect of anti-CD40 in combination with RT and CTLA4 inhibition on tumor growth.
(a) Treatment schedule for CD8^{+} T cells depletion in 4T1 implanted mice treated with RT + CTLA4i + anti-CD40 (n=11 biologically independent mice per group). **(b)** Differential tumor volume between day 10 and day 24 (following tumor inoculation). Data show maximum tumor response in the different groups. Treatment groups were compared by Mann Whitney tests on Log-transformed values for statistical significance. **(c)** Survival analysis or mice treated with RT + CTLA4i + anti-CD40 with or without CD8^{+} T cells depletion. Statistical significancy was tested by log-rank test. Source data are provided in the Source Data file.

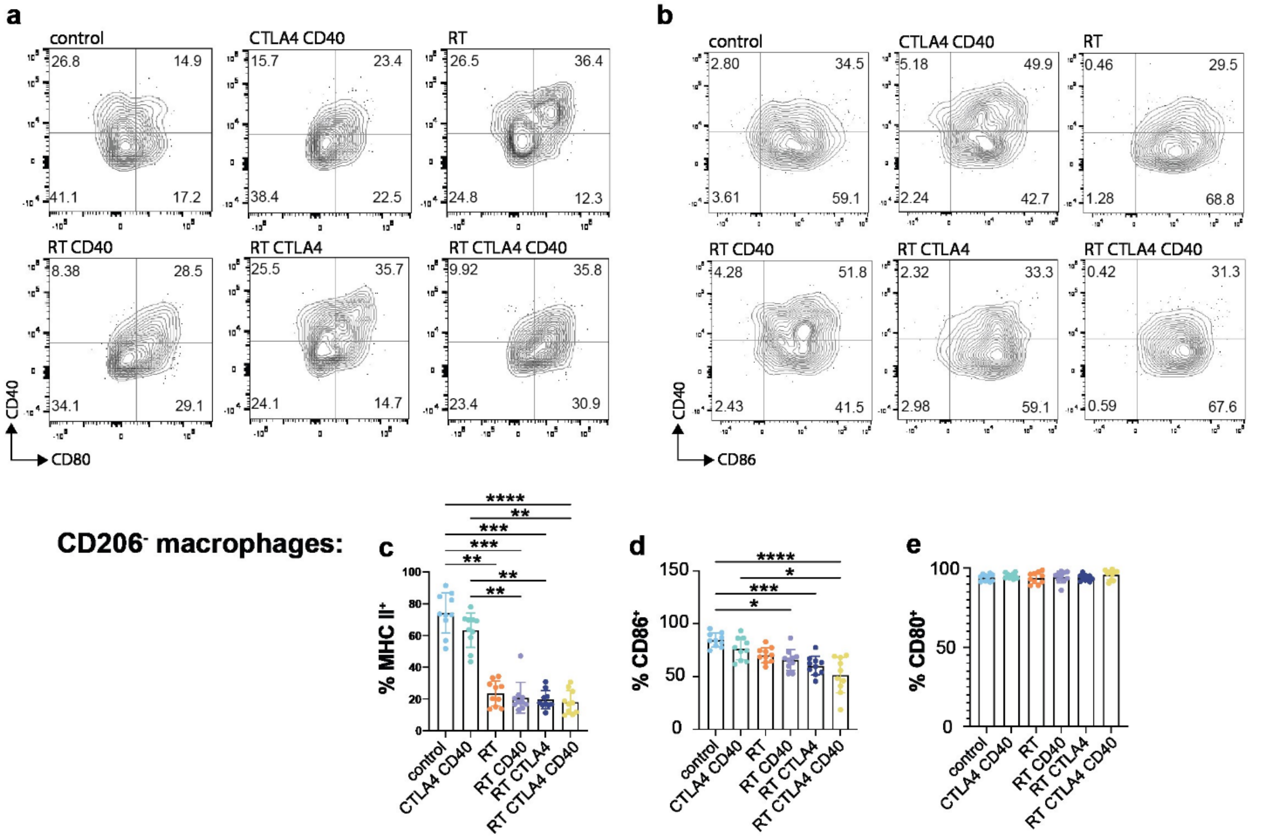

Supplementary Figure 19: Related to Figure 8.

(a, b) Total tumor infiltrated dendritic cells (defined as CD11b$^+$ f4/80$^-$ CD11c$^+$ MHC II$^+$) were down sampled (n=300 per sample/10 samples per groups) using the FlowJo DownSample (v 3.3.1) plugin and concatenated. Expression of CD40 and CD80 **(a)** and CD40 and CD86 **(b)** activation markers by the cDC1 (XCR1$^+$) population. Numbers indicate the percentage of cells in each quadrant. **(c-e).** Percentage of intratumoral CD206$^-$ macrophages expressing MHC II$^+$ **(c)**, CD86$^+$ **(d)**, and CD80$^+$ **(e)**. Data are shown as mean +/- SD, each dot represent one animal, n=10 per group. Populations were compared by Kruskal-Wallis and Dunn's post test for statistical significance. Statistical significance was assessed by Kruskal-Wallis test and post-hoc Dunn's test on each panel *, **, *** and ****, indicate p-values < 0.05, ≤ 0.01, <0,001 and <0,0001 respectively. Source data and exact p values are provided in the Source Data file.

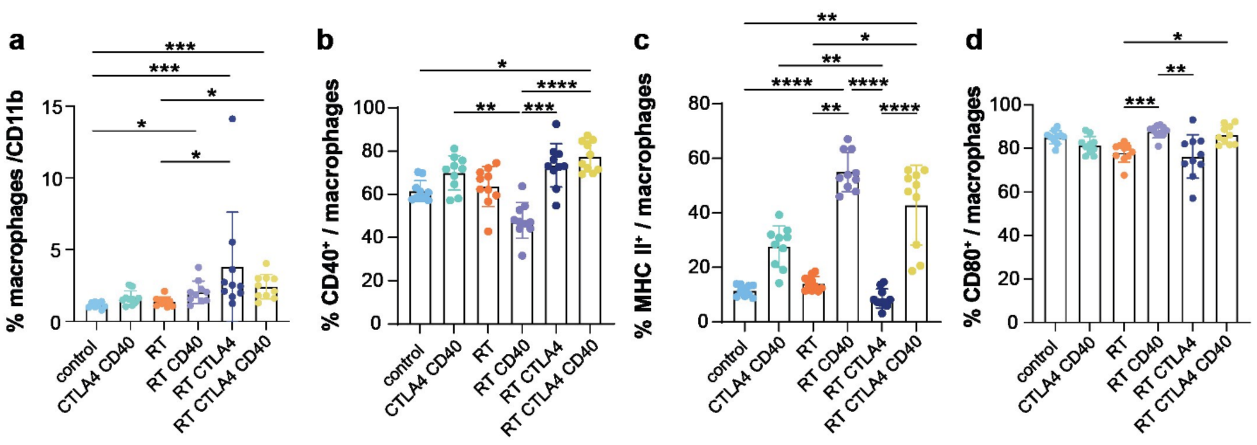

Supplementary Figure 20: Related to Figure 9.

(**a**) Percentage of macrophages defined as $CD11b^{+}$ $Ly6G^{-}$ $F4/80^{+}$ among $CD45^{+}$ lung cells; (**b**) CD40, (**c**) MHC II and (**d**) CD80 expression by lung macrophages. Data are shown as mean +/- SD, each dot represent one animal, n=10 biologically independent mice per group. Statistical significance was assessed by Kruskal-Wallis test and post-hoc Dunn's test on each panel. *, **, *** and ****, indicate p-values < 0.05, ≤ 0.01, <0,001 and <0,0001 respectively. Source data and exact p values are provided in the Source Data file.

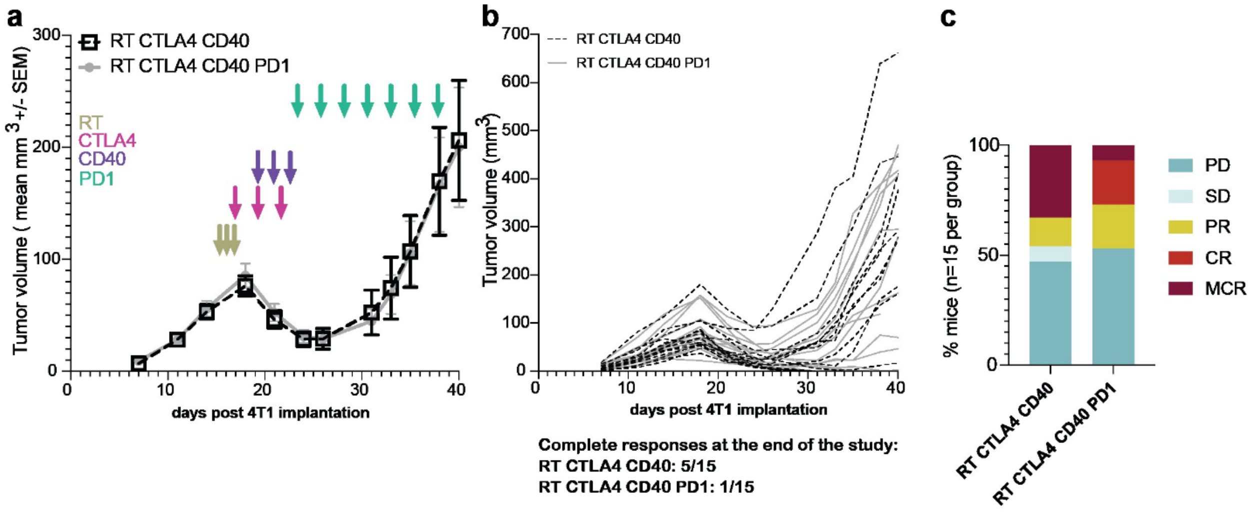

Supplementary Figure 21: PD1 blockade does not improve the anti-tumor effect of RT + CTLA4i + anti-CD40.

(a) Mean (+/- SEM) of tumor growth for mice treated with RT + CTLA4i + anti-CD40 with or without addition of anti-PD1 (n=15 biologically independent mice per group). Treatment schedule is indicated by colored arrows: RT: brown, CTLA4i: pink, anti-CD40: purple, anti-PD1: green. **(b)** Individual tumor growth curves for mice treated with RT+CTLA4+anti-CD40 (black dotted line) or RT+CTLA4i+anti-CD40+anti-PD1 (grey plain line). **(c)** Response to treatment of each mouse was measured using the 5-category method that classifies responses into maintained CR (MCR), CR, PR, SD, and PD. Source data are provided as a Source Data file.

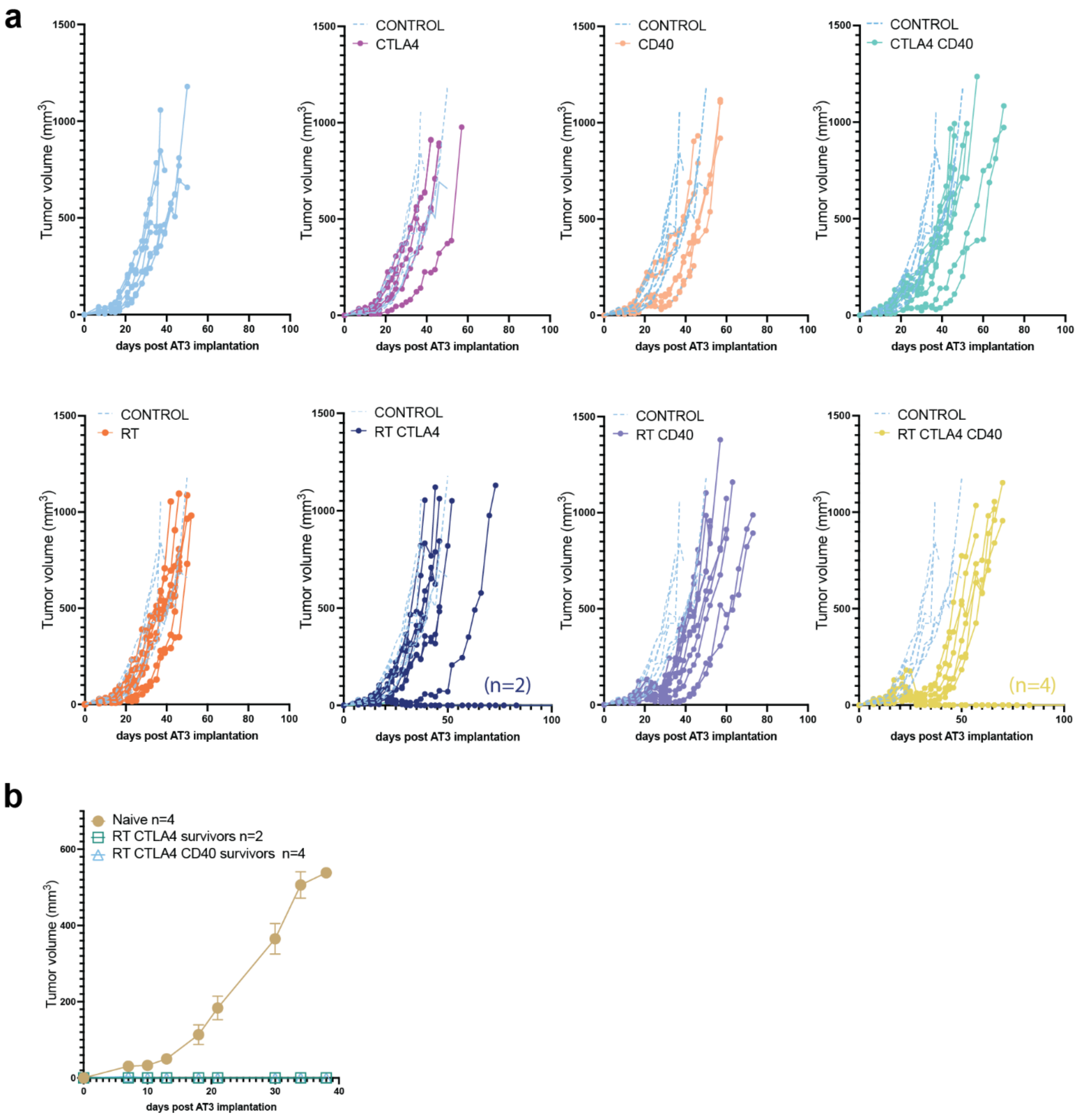

Supplementary Figure 22: AT3 Individual tumor growth curves, related to Figure 10.
(a) Individual tumor growth curves for mice treated with combination therapies as presented in figure 8a-d. Control (light blue) curves are provided in dotted lines on all panels as a comparison. The number of mice completely rejecting their tumor are indicated in each plot. (n=10 biologically independent mice per treatment group, n=5 for control group) **(b)** Tumor growth of long-term survivors rechallenged with AT3 cells in the opposite flank (n=2 for RT+CTLA4i, and n=4 for RT+CTLA4i+anti-CD40 group) compared to naïve mice (n=4). Data are shown as mean +/- SEM. Source data are provided as a Source Data file.

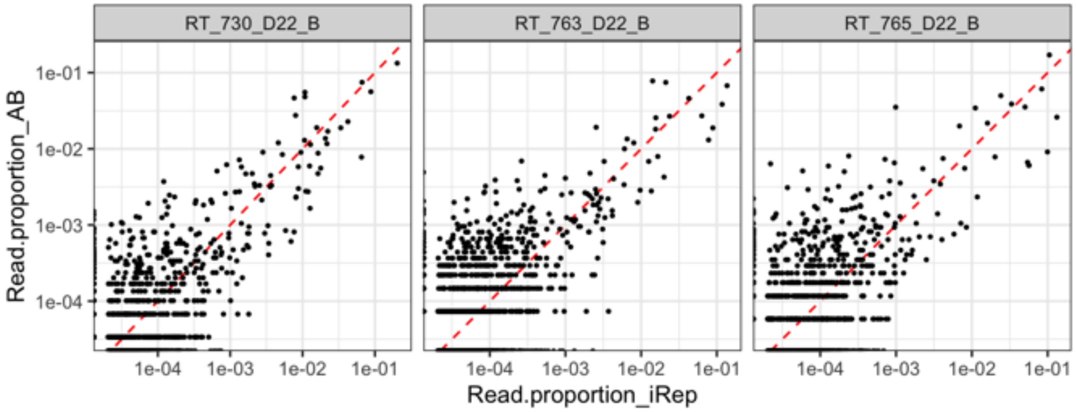

Supplementary Figure 23: Comparison of ImmunoSEQ and iRepertoire platforms. Frequency of T cell clonotypes of three tumors assessed with both Adaptive Biotech ImmunoSEQ and iRepertoire. The red dashed line indicates a diagonal line where the x-coordinate (proportion measured using the iRepertoire platform) equal the y-coordinate (proportion measured using Adaptive ImmunoSEQ platform). Source data are provided as a Source Data file.

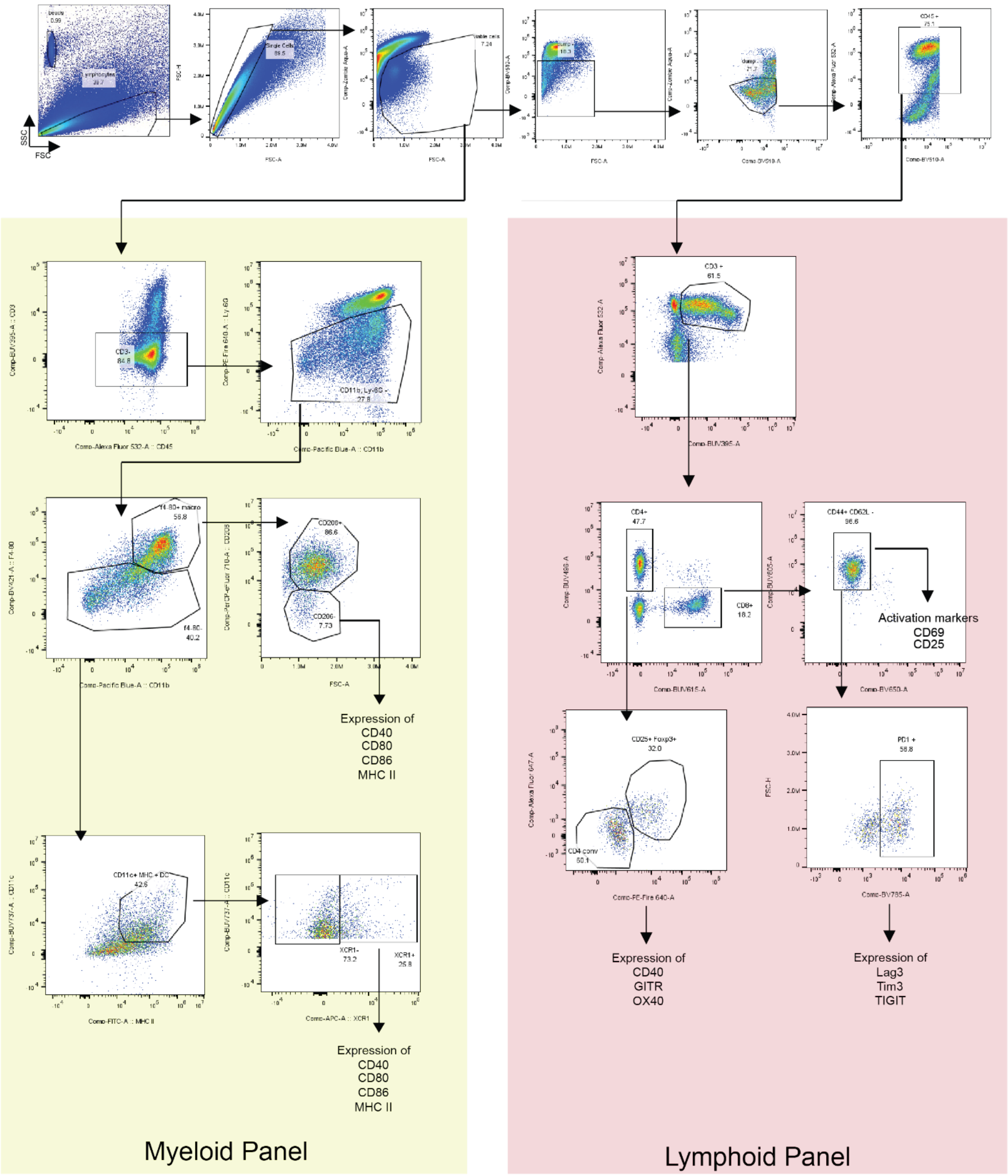

Supplementary Figure 24: Gating strategy applied to tumor immune infiltrate.
Gating strategy applied to 4T1 tumor samples stained with Panel 1 (lymphocytes – right panel) and Panel 2 (myeloid cells – left panel) detailed in supplementary tables S3 and S4. Manual gating was performed using FlowJo v.10 software. The lymphoid panel was used for Figure 5, and the myeloid panel for Figure 8.

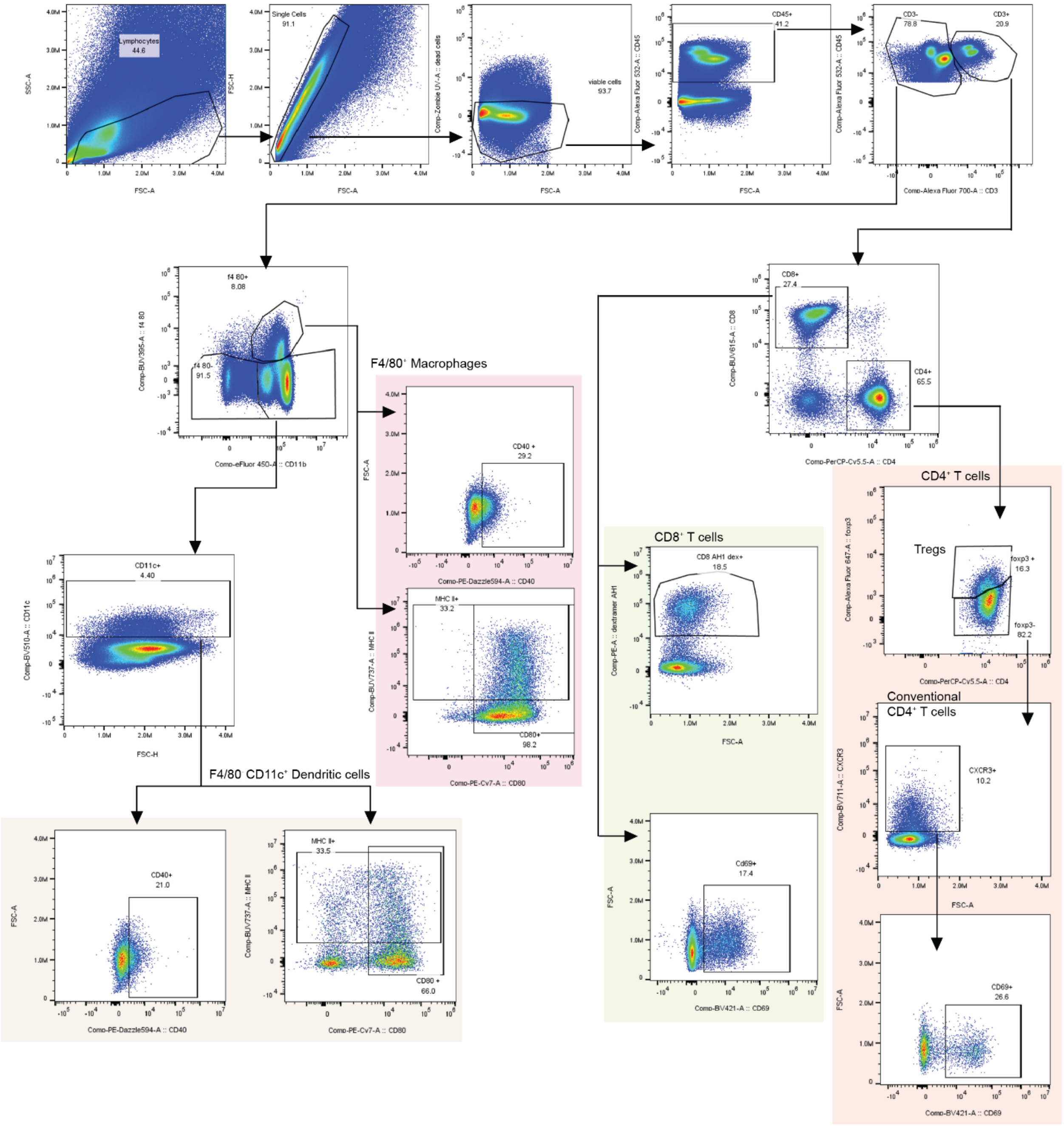

Supplementary Figure 25: **Gating strategy applied to lung immune infiltrate.**
Gating strategy applied to 4T1-bearing mice lung samples stained with the panel detailed in supplementary table S5. Manual gating was performed using FlowJo v.10 software. This panel was used for Figure 9.

Supplementary Tables

Cell subset	Change in CTLA4i treated tumorsa	Change in RT treated tumorsa	Change in RT+CTLA4i treated tumorsa
CD4 Naïve	(1.2%; log2(OR) = -1.0; p = 0.022)	(1.2%; log2(OR) = -0.9; p = 0.029)	(0.3%; log2(OR) = -3.2; p = 1.9e-08)
CD8 EA	(1.6%; log2(OR) = 0.5; p = 0.35)	(0.7%; log2(OR) = -0.8; p = 0.2)	(2.8%; log2(OR) = 1.3; p = 0.00053)
CD8 EM	(8.0%; log2(OR) = 0.4; p = 0.033)	(9.1%; log2(OR) = 0.6; p = 0.0011)	(16.5%; log2(OR) = 1.6; p = 5.6e-24)
CD8 Naïve	(3.3%; log2(OR) = -0.5; p = 0.036)	(2.0%; log2(OR) = -1.3; p = 4e-06)	(1.1%; log2(OR) = -2.1; p = 4.6e-11)
CD8 Tex	(30.4%; log2(OR) = -0.8; p = 7.4e-16)	(59.8%; log2(OR) = 1.0; p = 7.2e-24)	(35.5%; log2(OR) = -0.5; p = 2.7e-06)
CD8 Tpex	(3.6%; log2(OR) = 0.4; p = 0.17)	(1.7%; log2(OR) = -0.7; p = 0.036)	(5.9%; log2(OR) = 1.1; p = 5.1e-06)
Tfh	(1.0%; log2(OR) = 0.0; p = 1)	(0.1%; log2(OR) = -3.3; p = 0.00044)	(0.7%; log2(OR) = -0.4; p = 0.5)
Th1	(36.1%; log2(OR) = 1.1; p = 3.1e-24)	(9.8%; log2(OR) = -1.3; p = 3.9e-21)	(30.5%; log2(OR) = 0.7; p = 5.7e-11)
Treg	(14.7%; log2(OR) = -0.3; p = 0.022)	(15.6%; log2(OR) = -0.2; p = 0.11)	(6.6%; log2(OR) = -1.6; p = 8.4e-25)

Supplementary Table 1

Frequency of T cell subset in indicated group; log2 odds ratio of frequency compared to untreated tumors; FDR adjusted Fisher Exact test p-value of change, two-sided.

Cell subset	Change in CTLA4i treated tumors[a]	Change in RT treated tumors[a]	Change in RT+CTLA4i treated tumors[a]
CD8 EA in C1	(1.5%; log2(OR) = 0.8; p = 0.49)	(0.6%; log2(OR) = -0.4; p = 1)	(2.1%; log2(OR) = 1.4; p = 0.012)
CD8 EM in C1	(5.1%; log2(OR) = 0.7; p = 0.028)	(3.6%; log2(OR) = 0.2; p = 1)	(11.6%; log2(OR) = 2.0; p = 3.7e-22)
CD8 EM in C9	(0.9%; log2(OR) = -0.1; p = 1)	(2.8%; log2(OR) = 1.5; p = 0.00094)	(1.7%; log2(OR) = 0.8; p = 0.47)
CD8 Tex in C0	(18.1%; log2(OR) = -0.9; p = 1.7e-14)	(31.4%; log2(OR) = 0.1; p = 0.69)	(17.7%; log2(OR) = -0.9; p = 1.2e-15)
CD8 Tex in C1	(0.5%; log2(OR) = 0.0; p = 1)	(2.0%; log2(OR) = 2.1; p = 0.00023)	(2.4%; log2(OR) = 2.4; p = 6.6e-06)
CD8 Tex in C5	(4.1%; log2(OR) = -0.1; p = 1)	(8.3%; log2(OR) = 0.9; p = 3.4e-05)	(5.2%; log2(OR) = 0.2; p = 1)
CD8 Tex in C7	(2.8%; log2(OR) = 0.2; p = 1)	(5.6%; log2(OR) = 1.2; p = 3.4e-05)	(3.5%; log2(OR) = 0.5; p = 0.4)
CD8 Tex in C10	(2.1%; log2(OR) = -0.1; p = 1)	(4.1%; log2(OR) = 0.9; p = 0.011)	(1.9%; log2(OR) = -0.2; p = 1)
CD8 Tex in C12	(1.4%; log2(OR) = -0.3; p = 1)	(3.3%; log2(OR) = 1.0; p = 0.011)	(2.1%; log2(OR) = 0.4; p = 0.95)
CD8 Tpex in C6	(2.2%; log2(OR) = 0.4; p = 1)	(1.1%; log2(OR) = -0.6; p = 0.73)	(3.7%; log2(OR) = 1.1; p = 0.0025)
Th1 in C1	(1.7%; log2(OR) = 0.7; p = 0.59)	(0.5%; log2(OR) = -1.2; p = 0.26)	(2.8%; log2(OR) = 1.4; p = 0.002)
Th1 in C2	(3.6%; log2(OR) = 1.0; p = 0.014)	(1.6%; log2(OR) = -0.3; p = 1)	(1.4%; log2(OR) = -0.4; p = 1)
Th1 in C3	(16.2%; log2(OR) = 1.6; p = 6.4e-22)	(2.8%; log2(OR) = -1.2; p = 3.4e-05)	(15.2%; log2(OR) = 1.5; p = 1.4e-18)
Th1 in C4	(6.3%; log2(OR) = -0.2; p = 1)	(2.9%; log2(OR) = -1.3; p = 1.5e-07)	(3.9%; log2(OR) = -0.9; p = 0.00037)
Th1 in C11	(4.0%; log2(OR) = 1.1; p = 0.0016)	(1.0%; log2(OR) = -0.9; p = 0.26)	(2.6%; log2(OR) = 0.5; p = 0.68)
Treg in C2	(10.3%; log2(OR) = -0.3; p = 0.34)	(10.9%; log2(OR) = -0.2; p = 0.79)	(4.5%; log2(OR) = -1.6; p = 1.5e-16)
Treg in C8	(2.8%; log2(OR) = -0.5; p = 0.26)	(2.0%; log2(OR) = -1.1; p = 0.0025)	(1.4%; log2(OR) = -1.6; p = 6.6e-06)

Supplementary Table 2

Frequency of T cell subset in indicated group; log2 odds ratio of frequency compared to untreated tumors; FDR adjusted Fisher Exact test p-value of change, two-sided.

Cytek Aurora		Antibodies					Titration
Laser	Detector	Marker	Clone	Fluorochrome	Vendor	Reference	µg/ 100µl
355 nm	UV2	CD3	17A2	BUV395	BD	563565	0.5
355 nm	UV7	CD4	RM4-4	BUV496	BD	741051	0.3
355 nm	UV10	CD8	53-6.7	BUV615	BD	613004	0.1
355 nm	UV14	CD69	H1.2F3	BUV737	BD	612793	0.03
355 nm	UV16	CD44	IM7	BUV805	BD	741921	0.1
405 nm	V2	GITR	DTA-1	super bright 436	ebioscience	62-5874-82	0.03
405 nm	V7	Viability		Zombie Aqua	Biolegend	423101	
405 nm	V7	cd19	6D5	BV510	Biolegend	115545	0.2
405 nm	V7	Epcam	G8.8	BV510	Biolegend	118231	0.2
405 nm	V7	CD11b	M1/70	BV510	Biolegend	101245	0.2
405 nm	V7	CD11c	N418	BV510	Biolegned	117337	0.2
405 nm	V11	CD62L	MEL-14	BV650	Biolegend	104453	0.03
405 nm	V15	PD1	29F.1A12	BV785	Biolegend	135225	0.03
561 nm	YG4	CD25	PC61	PE fire 640	Biolegend	102071	0.03
488 nm	B3	CD45	30-F11	alexa fluor 532	eBioscience	58-0451-82	0.1
488 nm	B9	TIM3	5D12/TIM-3	BB700	BD	747619	0.3
561 nm	YG1	CD40Lg	SA047C3	PE	Biolegend	157003	2
561 nm	YG3	KI67	16A8	PE dazzle 594	Biolegend	652427	0.03
561 nm	YG9	OX40	OX-86	PE-Cy7	Biolegend	119415	0.03
640 nm	R2	FOXP3	MF23	alexa fluor 647	Biolegend	126407	0.1
640 nm	R4	CTLA4	UC10-4F10-11	APCR700	BD	565778	0.1
640 nm	R7	LAG3	eBioC9B7W (C9B7W)	APC eFluor780	ebioscience	47-223-180	0.1
488 nm	B10	TIGIT	GIGD7	PerCP eFluor 710	ebioscience	46-9501-80	0.3

Supplementary Table 3

Flow cytometry staining panel for intra-tumoral immune infiltrate staining (tumor panel 1). Detector V7 was used as a dump channel for dead and non-lymphoid cells.

Cytek Aurora		Antibodies					Titration
Laser	Detector	Marker	Clone	Fluorochrome	Vendor	Reference	µg/ 100µl
355 nm	UV2	CD3	145-2C11	BUV395	BD	563565	0.5
488 nm	B3	CD45	30-F11	alexa fluor 532	eBioscience	58-0451-82	0.1
405 nm	V7	Epcam	G8.8	BV510	Biolegend	118231	0.2
355 nm	UV14	CD11c	N418	BUV737	eBioscience	367-0114-80	0.5
405 nm	V3	CD11b	M1/70	Pacific blue	Biolegend	101224	0.03
405 nm	V1	F4/80	BM8	BV421	Biolegend	123137	0.3
561 nm	YG3	Ly6c	HK1.4	BV711	Biolegend	128037	0.03
561 nm	YG4	Ly6g	1A8	PE fire 640	Biolegend	127675	0.1
561 nm	YG1	CD40	3/23	PE	Biolegend	124610	1
488 nm	B2	MHC II	M5/114.15.2	FITC	ThermoFisher	11-5321-82	0.1
561 nm	YG9	CD80	16-10A1	PE Cy7	Biolegend	104734	0.3
640 nm	R7	CD86	GL1	APC Cy7	Biolegend	105029	0.3
640 nm	R1	XCR1	ZET	APC	Biolegend	148205	0.1
488 nm	B10	CD206	MR6F3	PerCP eFluor 710	ThermoFisher	46-2061-82	0.06
355 nm	UV7	Viability	-	Zombie UV	Biolegend	423107	

Supplementary Table 4

Flow cytometry staining panel for intra-tumoral myeloid infiltrate staining (tumor panel 2).

Cytek Aurora		Antibodies					Titration
**Laser**	**Detector**	**Marker**	**Clone**	**Fluorochrome**	**Vendor**	**Reference**	**µg/ 100µl**
640 nm	R4	CD3	17A2	alexa Fluor 700	eBioscience	56-0032-82	0.25
488 nm	B3	CD45	30-F11	alexa fluor 532	eBioscience	58-0451-82	0.1
488 nm	B9	CD4	GK1.5	Percp Cy5.5	Biolegend	100434	0.25
355 nm	UV10	CD8	53-6.7	BUV615	BD	613004	0.1
561 nm	YG4	CD25	PC61	PE fire 640	Biolegend	102071	0.03
405 nm	V1	CD69	H1.2F3	BV421	Biolegend	104545	0.4
640 nm	R2	Foxp3	MF23	alexa fluor 647	Biolegend	126407	0.1
405 nm	V15	PD1	29F.1A12	BV785	Biolegend	135225	0.03
561 nm	YG3	CXCR3	S18001A	BV711	Biolegend	155917	0.2
405 nm	V13	CD40	3 / 23	PE Dazzle 594	Biolegend	124629	0.4
405 nm	V7	CD11c	N418	BV510	Biolegend	117337	0.2
405 nm	V3	CD11b	M1/70	E fluor 450	eBioscience	48-0112-82	0.2
405 nm	V11	XCR1	ZET	BV650	Biolegend	148205	0.25
355 nm	UV2	F4/80	T45-2342	BUV395	BD	565614	0.5
355 nm	UV14	MHC II	M5/114.15.2	BUV737	BD	748845	0.2
561 nm	YG9	CD80	16-10A1	PE Cy7	Biolegend	104734	0.5
561 nm	YG1	dextramer AH1	-	PE	IMMUDEX	JG3294-PE	10µL
355 nm	UV7	Viability	-	Zombie UV	Biolegend	423107	

Supplementary Table 5

Flow cytometry staining panel for staining of immune cells in the lungs.
