# Supplementary material for: Immunotherapy targeting different immune compartments in combination with radiation therapy induces regression of resistant tumors
Source: Nat Commun. 2023 Aug 24;14:5146. doi: 10.1038/s41467-023-40844-3 (PMC10449830; doi:10.1038/s41467-023-40844-3)
Supplement: Supplementary file 3 — Description of Additional Supplementary Files [file 41467_2023_40844_MOESM3_ESM.pdf]

Description of Additional Supplementary Files

Supplementary Data 1

CDR3B sequences of AH1-specific TCRs defined in ref. 9 and in current manuscript.

Supplementary Data 2

Differentially expressed genes for ProjecTILs-defined subsets within each Seurat cluster (all genes)

Supplementary Data 3

Differentially expressed genes for ProjecTILs-defined subsets within each Seurat cluster (genes passing differential expression threshold of log2 fold change > 1, adjusted p-value > 0.01, and percent in target single cell population > 60%)

Supplementary Data 4

Metadata associated with 4T1 tumor RNAseq and deposited in SRA bioproject PRJNA596248. Related to Figures 2 and 7.

Supplementary Data 5

Annotated sample information for iRepertoire TCR sequencing deposited in SRA bioproject PRJNA596248.
Related to Figure 1.

Supplementary Data 6

Annotated sample information for Adaptive TCR sequencing in tumors and lymph nodes deposited into the ImmuneACCESS project repository of the Adaptive Biotechnology database (https://doi.org/10.21417/NR2023NC).
Related to Figure 7.

Supplementary Data 7

Annotated sample information for Adaptive TCR sequencing for three mice (ID#: RT_730_D22; RT_763_D22; RT_765_D22) deposited into the ImmuneACCESS project repository of the Adaptive Biotechnology database (https://doi.org/10.21417/NR2023NC) sequenced also with iRepertoire (iRepertoire data are in Supplementary Data 5 and are deposited in SRA bioproject PRJNA596248). Related to Figure S23.
