# Supplementary material for: Immunotherapy targeting different immune compartments in combination with radiation therapy induces regression of resistant tumors
Source: Nat Commun. 2023 Aug 24;14:5146. doi: 10.1038/s41467-023-40844-3 (PMC10449830; doi:10.1038/s41467-023-40844-3)
Supplement: Supplementary file 11 — Reporting Summary [file 41467_2023_40844_MOESM11_ESM.pdf]

nature portfolio

Corresponding author(s): Sandra Demaria

Last updated by author(s): Aug 11, 2023

Reporting Summary

Nature Portfolio wishes to improve the reproducibility of the work that we publish. This form provides structure for consistency and transparency in reporting. For further information on Nature Portfolio policies, see our Editorial Policies and the Editorial Policy Checklist.

Statistics

For all statistical analyses, confirm that the following items are present in the figure legend, table legend, main text, or Methods section.

n/a	Confirmed	
☐	☒	The exact sample size (*n*) for each experimental group/condition, given as a discrete number and unit of measurement
☐	☒	A statement on whether measurements were taken from distinct samples or whether the same sample was measured repeatedly
☐	☒	The statistical test(s) used AND whether they are one- or two-sided<br>*Only common tests should be described solely by name; describe more complex techniques in the Methods section.*
☐	☒	A description of all covariates tested
☐	☒	A description of any assumptions or corrections, such as tests of normality and adjustment for multiple comparisons
☐	☒	A full description of the statistical parameters including central tendency (e.g. means) or other basic estimates (e.g. regression coefficient) AND variation (e.g. standard deviation) or associated estimates of uncertainty (e.g. confidence intervals)
☐	☒	For null hypothesis testing, the test statistic (e.g. *F*, *t*, *r*) with confidence intervals, effect sizes, degrees of freedom and *P* value noted<br>*Give P values as exact values whenever suitable.*
☒	☐	For Bayesian analysis, information on the choice of priors and Markov chain Monte Carlo settings
☒	☐	For hierarchical and complex designs, identification of the appropriate level for tests and full reporting of outcomes
☒	☐	Estimates of effect sizes (e.g. Cohen's *d*, Pearson's *r*), indicating how they were calculated

Our web collection on statistics for biologists contains articles on many of the points above.

Software and code

Policy information about availability of computer code

Data collection	Qubit 3.0 system<br>MiSeq Reagent Kit v3, 600 Cycles, Illumina<br>HiSeq Rapid SBS Kit v2, Illumina<br>10x Genomics, PN-2000119 and PN-1000071<br>Agilent Bioanalyzer 2100<br>Cytek Aurora spectral flow cytometry platform with a 5 lasers (16UV-16V-14B-10YG-8R) fixed configuration
Data analysis	Illumina bcl2fastq2 v2.20 Conversion Software<br>Cutadapt v1.9.1<br>STAR v2.5.2b<br>HTSeq-count v0.11.2<br>DESeq2 v1.22.2<br>Rstudio Open Source edition<br>divo, tcR, immunarch and tidyverse packages in Rv3.5.2<br>Illumina's Real Time Analysis software (RTA) v3.4.4<br>10x Genomics Cell Ranger Single Cell Software suite v3.0.2<br>Seurat v3.0.2 in R v3.5.2<br>Survival v2.44.1.1 and survminer v0.4.6

FlowJo v10
DownSample v3.3.1
FlowSOM v3.0.18 plugins
cloud-based analysis platform OMIQ (https://omiq.ai)

For manuscripts utilizing custom algorithms or software that are central to the research but not yet described in published literature, software must be made available to editors and reviewers. We strongly encourage code deposition in a community repository (e.g. GitHub). See the Nature Portfolio guidelines for submitting code & software for further information.

Data

Policy information about availability of data

All manuscripts must include a data availability statement. This statement should provide the following information, where applicable:
- Accession codes, unique identifiers, or web links for publicly available datasets
- A description of any restrictions on data availability
- For clinical datasets or third party data, please ensure that the statement adheres to our policy

Raw RNA sequencing data generated during the current study have been deposited in NCBI's Sequence Read Archive (SRA) database under the accession number of PRJNA596248 [https://www.ncbi.nlm.nih.gov/sra/?term=PRJNA596248] and sample annotation is available in Supplementary Data 4 for RNAseq, and Supplementary Data 5 for TCRseq. CDR3B sequences of AH1-specific TCRs are listed in Supplementary Data 1. The raw DNA TCR sequence data have been deposited into the ImmuneACCESS project repository of the Adaptive Biotechnology database [https://doi.org/10.21417/NR2023NC], and sample annotation is available in Supplementary Data 6 and 7. Transcriptome profiling data available for breast cancer patients were downloaded from the publicly available METABRIC and The Cancer Genome Atlas (TCGA) databases from the http://www.cbioportal.org portal along with corresponding clinical information. The remaining data presented in the manuscript are available within the Source Data file and Supplementary Data 2 and 3. Source data are provided with this paper.

Human research participants

Policy information about studies involving human research participants and Sex and Gender in Research.

Reporting on sex and gender	This study does not include human subjects
Population characteristics	N/A
Recruitment	N/A
Ethics oversight	N/A

Note that full information on the approval of the study protocol must also be provided in the manuscript.

Field-specific reporting

Please select the one below that is the best fit for your research. If you are not sure, read the appropriate sections before making your selection.

☒ Life sciences ☐ Behavioural & social sciences ☐ Ecological, evolutionary & environmental sciences

For a reference copy of the document with all sections, see nature.com/documents/nr-reporting-summary-flat.pdf

Life sciences study design

All studies must disclose on these points even when the disclosure is negative.

Sample size	Sample size was calculated based on previous experiments that employed sequencing (Rudqvist et al., CIR 2018) with the adjustment of including paired pre and post therapy samples within each mouse. Comparisons were made between pre vs. pre, pre vs. post, and post vs. post. For therapeutic experiment testing, sample sizes were based on previous experiments in which a therapeutically relevant effect size was statistically significant.
Data exclusions	No data were excluded.
Replication	All experiments were repeated at least once, but each experiment involving mice was designed to provide additional information, in accordance with the three Rs (replacement, reduction, refinement) principle of humane use of vertebrate animals in research to maximize the information obtained per animal.<br>All attempts to replicate the results were successful.
Randomization	Mice were randomized in R for all experiments before start of treatment. Randomization was not relevant for in vitro experiment (Supplementary Figure 2) in which each experimental group was treated under the same conditions.
Blinding	Investigators that measured tumor growth were not blinded re: which treatment group each mouse belonged to because the same person

performed both tasks. Investigators were blinded during the analysis of gene expression and flow cytometry data.

Reporting for specific materials, systems and methods

We require information from authors about some types of materials, experimental systems and methods used in many studies. Here, indicate whether each material, system or method listed is relevant to your study. If you are not sure if a list item applies to your research, read the appropriate section before selecting a response.

Materials & experimental systems

n/a	Involved in the study
☐	☒ Antibodies
☐	☒ Eukaryotic cell lines
☒	☐ Palaeontology and archaeology
☐	☒ Animals and other organisms
☒	☐ Clinical data
☒	☐ Dual use research of concern

Methods

n/a	Involved in the study
☒	☐ ChIP-seq
☐	☒ Flow cytometry
☒	☐ MRI-based neuroimaging

Antibodies

Antibodies used

1- InVivoPlus anti-mouse CTLA-4, BioXCell, Cl: 9H10, cat #BP0131, 10mg/kg - every 3 days
2- InVivoMab anti-mouse GITR, BioXCell, Cl: DTA-1, cat #BE0063, 50mg/kg - every 10 days
3- InVivoMab anti-mouse OX40, BIoXCell, Cl: OX-86, cat #BE0031, 10mg/kg - every 4 days
4- InVivoPlus anti-mouse PD-1, BioXCell, Cl: RMP1-14, cat #BP0146, 10mg/kg - every 3 days
5- InVivoMab anti-mouse LAG-3, BioXCell, Cl: C9B7W, cat #BE0174, 12,5mg/kg - every 3 days
6- InVivoMab anti-mouse CD40, BioXCell, Cl:FGK4.5, cat #BE0016-2, 5mg/kg - every other day
7- InVivoPlus anti-mouse CD8alpha, BioXCell, Cl:2.43, cat #BP0016, 10mg/kg - every 7 days
8- anti-mouse CD3, BD, Cl:17A2, BUV395 conjugated, cat 563565, lot 2146085, 0.5µg/100µl
9- anti-mouse CD4, BD, Cl: RM4-4, BUV496 conjugated, cat 741051, lot 2021883, 0.3µg/100µl
10- anti-mouseCD8, BD, Cl: 53-6.7, BUV615 conjugated, cat 613004, lot 1210174, 01µg/µl
11- anti-mouse CD69, Bd, Cl: H1.2F3, BUV737 conjugated, cat 612793, lot 1159821, 0.03µg/100µl
12- anti-mouse CD44, BD, Cl: IM7, BUV805 conjugated, cat 741921, lot 2021882, 0.1µg/100µl
13- anti-mouse XCR1, BioLegend Cl ZET, APC conjugated, cat 148205 lot B271768, 0.1µg/100µl
14- anti-mouse GITR, ebioscience, Cl: DTA-1, superBright 436 conjugated, cat 62-5874-82, lot 2296227, 0.03µg/100µl
15- anti mouse CD19, BioLegend, Cl: 6D5, BV510 conjugated, cat 115545, lot b335586, 0.2µg/100µl
16- anti-mouse Epcam, Biolegend, Cl: G8.8, BV510 conjugated, cat 118231, lot b346195, 0.2µg/100µl
17- anti-mouse CD11b, Biolegend, Cl: M1/70, BV510 conjugated, cat 101245, lot b360991, 0.2µg/100µl
18- anti-mouse CD11c, BioLegend, Cl: N418, BV510 conjugated, cat 117337, lot b352283, 0.2µg/100µl
19- anti-mouse CD62L, BioLegend, Cl: MEL-14, BV650 conjugated, cat 104453, lot b324616, 0.03µg/100µl
20- anti-mouse CD206, ThermoFisher, Cl: MR6F3, PerCP eFluor 710 conjugated, cat 46-2061-82, lot 2410786 0.06µg/100µl
21- anti-mouse PD1, BioLegend, Cl: 29F.1A12, BV785 conjugated, cat 135225, lot b337077, 0.03µg/100µl
22- anti-mouse CD25, BioLegend, Cl: PC61, PE Fire 640 conjugated, cat 102071, lot b333678, 0.03µg/100µl
23- anti-mouse CD45, eBioscience, Cl: 30-F11, alexa-fluor532 conjugated, cat 58-0451-82, lot 2442204, 0.1µg/100µl
24- anti-mouse TIM3, BD, Cl: 5D12/TIM3, BB700 conjugated, cat 747619, lot 2021880, 0.3µg/100µl
25- anti-mouse CD40Lg, BioLegend, Cl: SA047C3, PE conjugated, cat 157003, lot b330339, 2µg/100µl
26- anti-mouse Ki67, BioLegend, Cl: 16A8, PE Dazzle594 conjugated, cat 652427, lot b322606, 0.03µg/100µl
27- anti-mouse OX40, BioLegend, Cl: OX-86, PE-Cy conjugated, cat 119415, lot 324920, 0.03µg/100µl
28- anti-mouse FOXP3, BioLegend, Cl:MF23, alexa-fluor 647 conjugated, cat 126407, lot b343829, 0.1µg/100µl
29- anti-mouse CTLA4, BD, Cl: UC10-4F10-11, APC-R700 conjugated, cat 565778, lot 1294321, 0.1µg/100µl
30- anti-mouse LAG3, eBioscience, Cl: eBioC9B7W, APC eFluor780 conjugated, cat 47-223-180, lot 2413166, 0.1µg/100µl
31- anti-mouse TIGIT, eBioscience, Cl: GIGD7, PerCP eFluor 710 conjugated, cat 46-9501-80, lot 2433032/2452742, 0.3µg/100µl
32- anti-mouse CD3, eBioscience, Cl: 17A2, alexa-fluor 700 conjugated, cat 56-0032-82, lot E08933-1630, 0,25µg/100µl
33- anti-mouse CD4, BioLegend, Cl: GK1.5, PerCP-Cy5.5 conjugated, cat 100434, lot B276680, 0.1µg/100µl
34- anti-mouse CD69, BioLegend, Cl: H1.2F3, BV421 conjugated, cat 104545, 0.4µg/100µl
35- anti-mouse CXCR3, BioLegend, Cl: S18001A, BV711 conjugated, cat 155917, 0.2µg/100µl
36- anti-mouse CD40, BioLegend, Cl: 3/23, PE Dazzle594 conjugated, cat 124629, 0.4µg/100µl
37- anti-mouse CD11b, eBioscience, Cl: M1/70, eFluor 450 conjugated, cat 48-0112-82, lot 2198693, 0.2µg/100µl
38- anti-mouse XCR1, BioLegend, Cl: ZET, BV650 conjugated, cat 148205, lot b251929, 0.25µg/100µl
39- anti-mouse F4/80, BD, Cl: T45-2342, BUV395 conjugated, cat 565614, lot 1104580, 0.5µg/100µl
40- anti-mouse MHC II, BD, Cl: M5/114.15.2, BUV737 conjugated, cat 748845, lot 1188121, 0.2µg/100µl
41- anti-mouse CD80, BioLegend, Cl: 16-10A1, PE Cy7 conjugated, cat 104734, lot b277123, 0.5µg/100µl
42- anti-mouse CD3, BD, Cl: 145-2C11, BUV395 conjugated, cat 563565, lot 2146085, 0.5µg/100µl
43- anti-mouse CD11c, eBioscience, Cl: N418, BUV737 conjugated, cat 367-0114-80 lot 2599059, 0.5µg/100µl
44- anti-mouse CD11b, BioLegend, Cl: M1/70, Pacific Blue conjugated, cat 101224, lot B308096, 0.03µg/100µl
45- anti mouse f4/80, BioLegend, Cl: BM8, BV421 conjugated, cat 123137, lot B330997, 0.3µg/100µl

46- anti-mouse Ly6c, BioLegend, Cl: HK1.4, BV711 conjugated, cat 128037, lot B345240, 0.03µg/100µl
47- anti-mouse Ly6g, BioLegend, Cl: 1A8, PE Fire 640 conjugated, cat 127675, lot B359619, 0.1µg/100µl
48- anti-mouse CD40, BioLegend, Cl: 3/23, PE conjugated, cat 124610, lot B353788 , 1µg/100µl
49- anti-mouse MHC II, ThermoFisher, Cl: M5/114.15.2, FITC conjugated, cat 11-5321-82, lot 2442242, 0.1µg/100µl
50- anti-mouse CD80, BioLegend, Cl: 16-10A1, PE Cy7 conjugated, cat 104734, lot B367021, 0.3µg/100µl
51- anti-mouse CD86, BioLegend, Cl: GL1, APC Cy7 conjugated, cat 105029, lot B349514, 0.3µg/100µl

Validation

All antibodies used are commercially available and validated by manufacturers, with related data shown on the manufacturers websites.

For in vivo used antibodies:
1-https://bioxcell.com/invivoplus-anti-mouse-ctla-4-cd152-bp0131
2-https://bioxcell.com/invivomab-anti-mouse-gitr-be0063; Nat Med, 2019. 25(5): p. 759-766.
3-https://bioxcell.com/invivomab-anti-mouse-ox40-cd134-be0031; J Exp Med, 2008. 205(4): p. 825-39.
4-https://bioxcell.com/invivoplus-anti-mouse-pd-1-cd279-bp0146; Clin Cancer Res, 2017. 23(20): p. 6165-6177.
5-https://bioxcell.com/invivomab-anti-mouse-lag-3-be0174; Clin Cancer Res, 2017. 23(20): p. 6165-6177.
6-https://bioxcell.com/invivomab-anti-mouse-cd40-be0016-2; Cancer. Cancer Res, 2018. 78(15): p. 4282-4291.
7-https://bioxcell.com/invivoplus-anti-mouse-cd8a-bp0061 CD8 T cell depletion protocol was optimized in BALBc mice.

Flow cytometry antibodies were individually titrated and tested on mouse splenocytes and mouse tumor cell suspensions before use in the experiments. Antibody concentration was provided by the vendor for each lot:
8-https://www.bdbiosciences.com/en-us/products/reagents/flow-cytometry-reagents/research-reagents/single-color-antibodies-ruo/buv395-hamster-anti-mouse-cd3e.563565
9-https://www.bdbiosciences.com/en-us/products/reagents/flow-cytometry-reagents/research-reagents/single-color-antibodies-ruo/buv496-rat-anti-mouse-cd4.741051
10-https://www.bdbiosciences.com/en-us/products/reagents/flow-cytometry-reagents/research-reagents/single-color-antibodies-ruo/buv615-rat-anti-mouse-cd8a.613004
11-https://www.bdbiosciences.com/en-us/products/reagents/flow-cytometry-reagents/research-reagents/single-color-antibodies-ruo/buv737-hamster-anti-mouse-cd69.612793
12-https://www.bdbiosciences.com/en-us/products/reagents/flow-cytometry-reagents/research-reagents/single-color-antibodies-ruo/buv805-rat-anti-mouse-cd44.741921
13-https://www.biolegend.com/en-us/products/apc-anti-mouse-rat-xcr1-antibody-10222
14-https://www.thermofisher.com/antibody/product/CD357-AITR-GITR-Antibody-clone-DTA-1-Monoclonal/62-5874-82
15-https://www.biolegend.com/en-us/products/brilliant-violet-510-anti-mouse-cd19-antibody-8563
16-https://www.biolegend.com/en-us/products/brilliant-violet-510-anti-mouse-cd326-ep-cam-antibody-13764
17-https://www.biolegend.com/en-us/products/brilliant-violet-510-anti-mouse-human-cd11b-antibody-7993
18-https://www.biolegend.com/en-us/products/brilliant-violet-510-anti-mouse-cd11c-antibody-8491
19-https://www.biolegend.com/en-us/products/brilliant-violet-650-anti-mouse-cd62l-antibody-17377
20-https://www.thermofisher.com/antibody/product/CD206-MMR-Antibody-clone-MR6F3-Monoclonal/46-2061-82
21-https://www.biolegend.com/en-us/products/brilliant-violet-785-anti-mouse-cd279-pd-1-antibody-9874
22-https://www.biolegend.com/en-us/products/pe-fire-640-anti-mouse-cd25-antibody-20715
23-https://www.thermofisher.com/antibody/product/CD45-Antibody-clone-30-F11-Monoclonal/58-0451-82
24-https://www.bdbiosciences.com/en-us/products/reagents/flow-cytometry-reagents/research-reagents/single-color-antibodies-ruo/bb700-mouse-anti-mouse-cd366-tim-3.747619
25-https://www.biolegend.com/en-us/products/pe-anti-mouse-cd154-cd40l-antibody-18386
26-https://www.biolegend.com/en-us/products/pe-dazzle-594-anti-mouse-ki-67-antibody-13822
27-https://www.biolegend.com/en-us/products/pe-cyanine7-anti-mouse-cd134-ox-40-antibody-12101
28-https://www.biolegend.com/en-us/products/alexa-fluor-647-anti-mouse-foxp3-antibody-4662
29-https://www.bdbiosciences.com/en-us/products/reagents/flow-cytometry-reagents/research-reagents/single-color-antibodies-ruo/apc-r700-hamster-anti-mouse-cd152.565778
30-https://www.thermofisher.com/antibody/product/CD223-LAG-3-Antibody-clone-eBioC9B7W-C9B7W-Monoclonal/47-2231-80
31-https://www.thermofisher.com/antibody/product/TIGIT-Antibody-clone-GIGD7-Monoclonal/46-9501-80
32-https://www.thermofisher.com/antibody/product/CD3-Antibody-clone-17A2-Monoclonal/56-0032-82
33-https://www.biolegend.com/en-us/products/percp-cyanine5-5-anti-mouse-cd4-antibody-4220
34-https://www.biolegend.com/en-us/products/brilliant-violet-421-anti-mouse-cd69-antibody-7358
35-https://www.biolegend.com/en-us/products/brilliant-violet-711-anti-mouse-cd183-cxcr3-antibody-20690
36-https://www.biolegend.com/en-us/products/pe-dazzle-594-anti-mouse-cd40-antibody-13040
37-https://www.thermofisher.com/antibody/product/CD11b-Antibody-clone-M1-70-Monoclonal/48-0112-82
38-https://www.biolegend.com/en-us/products/apc-anti-mouse-rat-xcr1-antibody-10222
39-https://www.bdbiosciences.com/en-us/products/reagents/flow-cytometry-reagents/research-reagents/single-color-antibodies-ruo/buv395-rat-anti-mouse-f4-80.565614
40-https://www.bdbiosciences.com/en-us/products/reagents/flow-cytometry-reagents/research-reagents/single-color-antibodies-ruo/buv737-rat-anti-mouse-i-a-i-e.748845
41-https://www.biolegend.com/en-us/products/pe-cyanine7-anti-mouse-cd80-antibody-9320
42-https://www.bdbiosciences.com/en-us/products/reagents/flow-cytometry-reagents/research-reagents/single-color-antibodies-ruo/buv395-hamster-anti-mouse-cd3e.563565
43-https://www.thermofisher.com/antibody/product/CD11c-Antibody-clone-N418-Monoclonal/367-0114-80
44-https://www.biolegend.com/en-us/products/pacific-blue-anti-mouse-human-cd11b-antibody-3863
45-https://www.biolegend.com/en-us/products/brilliant-violet-421-anti-mouse-f4-80-antibody-7199

46-https://www.biolegend.com/en-us/products/brilliant-violet-711-anti-mouse-ly-6c-antibody-8935
47-https://www.biolegend.com/en-us/products/pe-fire-640-anti-mouse-ly-6g-antibody-22238
48-https://www.biolegend.com/en-us/products/pe-anti-mouse-cd40-antibody-4983
49-https://www.thermofisher.com/antibody/product/MHC-Class-II-I-A-I-E-Antibody-clone-M5-114-15-2-Monoclonal/11-5321-82
50-https://www.biolegend.com/en-us/products/pe-cyanine7-anti-mouse-cd80-antibody-9320
51-https://www.biolegend.com/en-us/products/apc-cyanine7-anti-mouse-cd86-antibody-6554

Eukaryotic cell lines

Policy information about cell lines and Sex and Gender in Research

Cell line source(s)	4T1 cells were obtained from Fred R. Miller of Karmanos Cancer Institute<br>AT-3 cells were obtained from Jeffrey Schlom (NIH)
Authentication	Cell lines were authenticated by IDEXX Bioresearch in 2019 and 2016 respectively, by genetic evaluation of interspecies contamination and mouse STR profile.
Mycoplasma contamination	Cell lines were regularly tested for mycoplasma contamination and confirmed to be negative.
Commonly misidentified lines (See ICLAC register)	No commonly misidentified cell lines were used in this study

Animals and other research organisms

Policy information about studies involving animals; ARRIVE guidelines recommended for reporting animal research, and Sex and Gender in Research

Laboratory animals	BALB/cAnN (Stock #: BALB) from Taconic Farms - Wildtype - 6-8 weeks females<br>C57BL/6J (Stock #: 000664) from JACKSON LABORATORY - Wildtype - 6-8 weeks females were used in this study. The animal holding room is maintained at 72 ± 2 °F (21.5 ± 1 °C), relative humidity between 30% and 70%, and a 12:12 hour light:dark photoperiod.
Wild animals	Study did not involve wild animals.
Reporting on sex	This study involves mouse models of triple negative breast cancer, thus the in vivo experiments were performed exclusively on females.
Field-collected samples	Study did not involve collection of samples from the field.
Ethics oversight	All in vivo experiments were approved by the Institutional Animal Care and Use Committee at Weill Cornell Medicine.

Note that full information on the approval of the study protocol must also be provided in the manuscript.

Flow Cytometry

Plots

Confirm that:

- [x] The axis labels state the marker and fluorochrome used (e.g. CD4-FITC).
- [x] The axis scales are clearly visible. Include numbers along axes only for bottom left plot of group (a 'group' is an analysis of identical markers).
- [x] All plots are contour plots with outliers or pseudocolor plots.
- [x] A numerical value for number of cells or percentage (with statistics) is provided.

Methodology

Sample preparation	Lungs were perfused by injection of 10mL cold PBS through the right ventricle prior to collection. Tumors and lungs were excised, chopped into small pieces, and enzymatically dissociated using Mouse Tumor Dissociation Kit (Miltenyi Biotec, cat #130-096-730) and Lung Dissociation Kit (Miltenyi Biotec, cat #130-095-927) on a gentleMACS Octo Dissociator (Miltenyi Biotec). Tissue homogenates were resuspended in 10% FBS – RPMI-1640 (Corning) and filtered on a 70µm strainer to remove large debris. Cells were washed in cold PBS. Lung cell suspension was resuspended in a working solution of red blood cell (RBC) Lysis Buffer (eBioscience, cat #00-4300-54) and incubated for 2min at room temperature. Cells were washed twice in PBS. Cells stained with AH1-dextramer PE (Immudex, cat #JG3294-PE) were pretreated with 50nM dasatinib (Sigma-Aldrich) for 30 min at 37ºC, before addition of the PE conjugated AH1-dextramer were added per test and cells were incubated on ice for 30 min and washed in PBS. Viability dye staining (Zombie Aqua, Biolegend cat #423101 or Zombie UV, Biolegend cat #423107) was performed prior to surface staining. Surface staining were done in Brilliant Stain Buffer (Thermo Fisher Scientific). Cells were fixed and permeabilized on ice using the Foxp3 / Transcription Factor Staining Buffer Set (eBioscience, cat #00-5523-00). Intra-cellular staining with Ki67 (cl 16A8; PE Dazzle 594) and/or FOXP3 (cl MF23; alexa fluor 647) was performed on ice.

Instrument	Cytek Aurora spectral flow cytometry platform with a 5 lasers (16UV-16V-14B-10YG-8R) configuration.
Software	Analysis were performed using FlowJo v10 software and cloud-based analysis platform OMIQ (https://omiq.ai).
Cell population abundance	For flow cytometry experiments, Fluorescence minus one (FMO) or fluorescence minus multiple (FMM) controls were used. At least 100000 events per sample were acquired
Gating strategy	Standard gating methods were applied taking into account unstained and FMO controls

☒ Tick this box to confirm that a figure exemplifying the gating strategy is provided in the Supplementary Information.
